# Suppressing the Hofmeister Anion Effect by Thermal Annealing of Thin-Film Multilayers Made of Weak Polyelectrolytes

**DOI:** 10.1021/acs.macromol.2c01517

**Published:** 2022-10-26

**Authors:** Tin Klačić, Klemen Bohinc, Davor Kovačević

**Affiliations:** † Division of Physical Chemistry, Department of Chemistry, Faculty of Science, University of Zagreb, Horvatovac 102a, 10000 Zagreb, Croatia; ‡ Faculty of Health Sciences, University of Ljubljana, Zdravstvena pot 5, 1000 Ljubljana, Slovenia

## Abstract

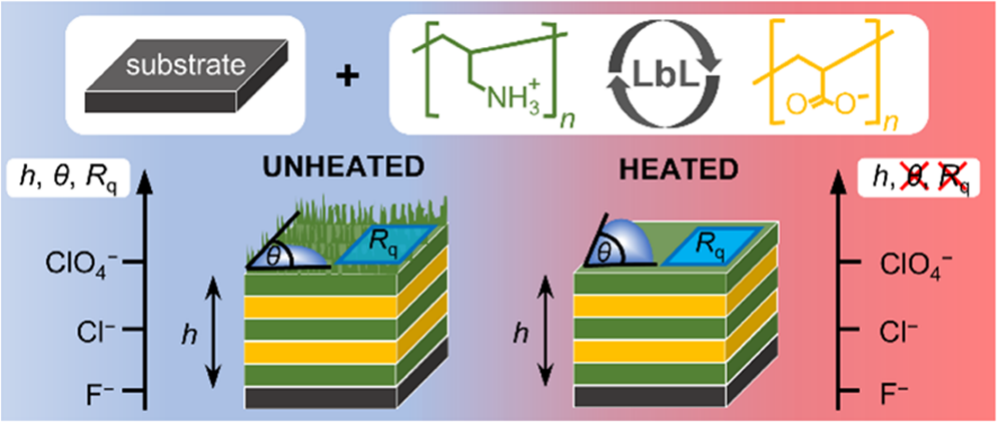

Thin films made of weak polyelectrolytes poly(allylamine hydrochloride) (PAH) and poly(acrylic acid) (PAA) have been fabricated on silicon wafers using the layer-by-layer (LbL) method. To study the influence of counteranion type on the growth and properties of PAH/PAA multilayers, the nature of the supporting sodium salt was varied from cosmotropic to chaotropic anions (F^–^, Cl^–^, and ClO_4_^–^). Results of ellipsometry and AFM measurements indicate that the film thickness and surface roughness systematically increase on the order F^–^ < Cl^–^ < ClO_4_^–^. Furthermore, we found that the hydrophobicity of the PAH/PAA multilayer also follows the described trend when a polycation is the terminating layer. However, the heating of PAH/PAA multilayers to 60 °C during the LbL assembly suppressed the influence of background anions on the multilayer formation and properties. On the basis of the obtained results, it could be concluded that thermal annealing induces changes at the polymer–air interface in the sense of reorientation and migration of polymer chains.

## 1 Introduction

Since Decher and co-workers introduced the layer-by-layer (LbL) method in the early 1990s, first to build up ultrathin multilayer films of bipolar amphiphiles^1^ and then of polyelectrolytes,^2^ this technique has been widespread in many laboratories. This simple method of alternate adsorption of positively and negatively charged macromolecules from solutions onto a charged surface has become the most common way of preparing polyelectrolyte multilayers (PEMs). These nanolayered polymeric systems are suitable for many applications including nanofiltration membranes,^3−5^ water-resistant coatings,^6^ microfluidic devices with superhydrophobic and superhydrophilic regions,^7^ antireflection and antifogging coatings,^8^ drug delivery systems,^9−12^ antibacterial coatings for urinary catheters,^13^ smart electronic fabrics for human biomonitoring,^14^ as well as strain and pH sensors.^15^ The wide application potential of PEMs lies in the fact that their structure and properties can be fine-tuned by varying experimental conditions such as pH,^16−18^ ionic strength,^18−21^ temperature,^22−24^ deposition time,^25^ and quality of the solvent.^25−27^

Besides the mentioned factors, the type of background salt also plays an important role in multilayer formation.^25,27−43^ Dubas and Schlenoff^25^ were among the first to observe that the thickness of LbL films depends on the cation and anion present in polyelectrolyte assembly solutions. They deposited 10 bilayers of sodium polystyrene sulfonate (PSS) and polydiallyldimethylammonium chloride (PDADMAC) on the silicon surface. Later on, Salomäki and co-workers^38^ conducted a more detailed study with that pair of strong polyelectrolytes. They found that surface roughness of the PDADMAC/PSS multilayer also depends on the type of the background salt. Salomäki and his collaborators further expanded their research^39^ and prepared several hundred PDADMAC/PSS layers on the quartz surface in 0.1 M solutions of different sodium salts. Analysis by a quartz crystal microbalance (QCM) showed that the type of anion affects not only the deposited mass of the polyelectrolyte but also the stiffness of the multilayer. Depending on the salt used, a significant difference in the stiffness of the film, from rubber-like to almost glass-like, has been achieved. In addition to the thickness, surface roughness, and stiffness of the polyelectrolyte multilayer, it was found that the coverage of the surface by the multilayer, i.e., film permeability,^28,40^ and swelling of the PEM in solution depend on the choice of the background electrolyte.^30,41,42,44−47^ Moreover, it was demonstrated that the biocompatibility of natural polysaccharide-based multilayers can be controlled by the type of supporting electrolytes.^32^

In various studies,^28,30,32,34,38,39,41−44^ it was found that for monovalent anions the properties of LbL films correlate well with the anion’s position in the Hofmeister series. Briefly, in 1888, Hofmeister^48^ reported that the type of supporting electrolyte affects the solubility of egg white proteins in water. Some ions precipitated proteins in water, while others helped solubilize them so Hofmeister arranged them in two series, one for anions and another for cations. For univalent anions, the series goes as follows: OH^–^ < F^–^ < HCOO^–^ < CH_3_COO^–^ < Cl^–^ < Br^–^ < NO_3_^–^ < I^–^ < SCN^–^ < ClO_4_^–^. In this series, often the chloride ion is treated as a median, while other anions are divided into two groups: cosmotropic ions or salting-out ions (left of chloride) and chaotropic ions or salting-in ions (right of chloride). Cosmotropic ions are small in size and polarizability. They have a high electric field at short distances and a well-ordered large hydration shell. On the contrary, chaotropic ions are large with significant polarizability. They have a weak electric field and a loose hydration shell that can be easily removed.^49^ It is worth mentioning that the Hofmeister series for monovalent cations similarly explains the salt dependence of PEM properties. However, the effect of cations is not so pronounced as for anions probably due to their smaller size and polarizability differences.^24,43^

Several attempts have been made to quantify the Hofmeister series, i.e., to correlate the ranking of ions in a series with a number of physical parameters. Although no such universal parameter has been found, the order of ions in the Hofmeister series was often associated with the ion’s polarizability, the ion’s hydration enthalpy and entropy, or the viscosity B coefficient of the Jones–Dole empirical expression.^38,39,42,43,50^ It should also be pointed out that ion-specific effects have been observed in many processes (e.g., aggregation of colloidal particles,^51−53^ surfactant adsorption,^54^ and phase behavior of the lipid system^55−57^), but the origin of these effects is still not fully understood. Nevertheless, univalent chaotropic anions, compared to cosmotropic ions, have a greater ability to screen the free charges of polycations in solution. As a result of charge screening, the percentage of extrinsic polyelectrolyte–counterion pairs increases and the polyelectrolyte adopts a more globular form. This leads to polyelectrolyte deposition in loopier conformations, and more polycation is needed in the surface charge compensation and thus thicker films with a higher surface roughness are formed.

Described ion-specific effects were mainly tested on multilayers prepared by the combination of a strong polyanion PSS and a strong PDADMAC or weak poly(allylamine hydrochloride) (PAH) polycation. As mentioned earlier, the Hofmeister series provides a general model for explaining the effects of monovalent anions on the properties of multilayers formed by this pair of polyelectrolytes. However, there are some exceptions. For instance, there is no influence of the supporting anions on the thickness of films made by the alternate deposition of poly(allylamine hydrochloride) and poly(sodium phosphates).^58^

The aim of this paper is to extend this type of research to multilayers made of weak polyelectrolytes. For this purpose, we used PAH as a model weak polycation and poly(acrylic acid) (PAA) as a model weak polyanion. We focus on the effect of the monovalent F^–^ and ClO_4_^–^ anions as representatives of cosmotropic and chaotropic ions, respectively, and, the neutral Cl^–^ anion as a median between these two groups. Ion specificity of these anions was explored in the content of film thickness, roughness, and wettability. Although the influence of salt type on PEM thickness and roughness has been a subject in several studies,^25,28−32,38,43^ one aspect that has not been adequately covered in previous reports is the effect of ions on film wettability. This paper is an attempt to study this property as a multilayer is assembled in different background electrolytes. Furthermore, the present investigation aims to go a step further by exposing the PAH/PAA films to mild heating during the buildup process to verify if the heat treatment would alter the PEM’s properties and influence the ion-specific effects.

## 2 Experimental Section

### 2.1 Materials

Single-side polished silicon wafers (⟨100⟩ orientation, P-doped with boron, 15 cm in diameter, Siltronic AG) of 0.7 mm thickness were cut to plates of approximative dimensions of 7 × 1 cm^2^ for contact angle and ellipsometric measurements or 1 × 1 cm^2^ for atomic force microscopy. The plates were then soaked in freshly prepared Piranha solution for about 1 h, rinsed thoroughly with deionized water, dried with a stream of argon gas (5.0, Messer), and stored under ambient conditions in a well-sealed plastic container. The Piranha solution was prepared as a 3:1 mixture of concentrated H_2_SO_4_ (Lach-Ner) and 30% H_2_O_2_ solution (Kemika). *Caution! Piranha solution is a very strong oxidizing agent and reacts violently with organic compounds. It should be handled with extreme care!* Poly(allylamine hydrochloride) (*M*_w_ ≈ 17 500 g/mol) and poly(acrylic acid) (*M*_n_ ≈ 130 000 g/mol) were used as received from Sigma-Aldrich. The monomer functionalization degrees (*f*), also known as degrees of substitution (DS),^59^ were determined by potentiometric titrations with a standardized NaOH solution (Titrisol, Merck). The values obtained were 0.88 ± 0.01 for PAH and 0.97 ± 0.02 for PAA. All polyelectrolyte solution concentrations were corrected according to *f*-values and are quoted with respect to the monomer repeating unit. Polymer solutions were made in a 3-(*N*-morpholino)propanesulfonic acid buffer (Sigma-Aldrich) and different salt environments. All salts (NaF, NaCl, and NaClO_4_) were purchased from Sigma-Aldrich. For precautions, polyelectrolytes and all salts were stored in a vacuum desiccator with anhydrous CaCl_2_ or silica gel as wetting agents due to hygroscopicity and/or reactivity with the atmosphere. Before dissolving them, PAH and PAA were dried at 60 °C and salts were dried at 110 °C for about 200 min. To ensure the maximum charge density of both polyions,^60^ the pH of polyelectrolyte–buffer–salt solutions was adjusted to 7.0 ± 0.1 with a 1.0 M NaOH solution (Titrisol, Merck). For that purpose, a pH meter (826 pH mobile, Metrohm) equipped with a combined glass microelectrode (6.0234.100, Metrohm), precalibrated with standard buffers (Fluka) of pH 3.0, 5.0, 7.0, and 9.0, was used. The water used in all experiments was prepared in a three-stage Millipore Milli-Q Plus 185 purification system and had an initial conductivity lower than 0.055 μS/cm.

### 2.2 Preparation of Polyelectrolyte Multilayers

Polyelectrolyte multilayers were prepared according to the layer-by-layer deposition method suggested by Decher and co-workers.^2^ The sequential dipping of the substrate in the polycation and polyanion solutions was carried out in the following way. The silicon plate was affixed to a steel shaft, and about three-quarters of the plate was immersed for 5 min in a 25 mL solution containing the polyelectrolyte (*c*_m_ = 0.01 M), the buffer (*c* = 0.01 M), and a sodium salt (*c* = 0.10 M). The solution was stirred with a magnetic stirrer (*v* ≈ 500 rpm) at room temperature during adsorption. After deposition, the coated substrate was immersed three times for 1 min in 25 mL of fresh deionized water and then was blow-dried with argon or nitrogen (Messer). At this step, some of the prepared LbL films were heated at 60 °C for about 30 min in a drier. The described LbL process was repeated for 10 cycles to yield a film of 10 layers. The first layer was always PAH so that an odd layer number indicates that the outermost layer is PAH, while an even layer number coincides with the PAA outermost layer. The prepared nanofilms are designated as (PAH/PAA)*_x_*, where the subscript *x* denotes the number of layer pairs in the assemblies.

### 2.3 Contact Angle Measurement

The static contact angle measurements were carried out with the Attension Theta T200-Basic Plus (Biolin Scientific) goniometer at (24 ± 2) °C and 30–50% relative humidity. Prior to each measurement set, the goniometer was calibrated with a 4.000 mm ± 1 μm tungsten carbide ball. Calibration, as well as experiments and data analysis, was done in the OneAttension computer program (version 3.2). Static advancing contact angle experiments were performed using a standard sessile drop method^61^ as follows. A drop of deionized water (*V* ≈ 5 μL) was placed on a sample by moving the tip vertically until contact was made between the water drop and the sample. Immediately after placing a droplet on a sample, images of the droplet (1216 pixel × 800 pixel) were taken for 10 s at a frequency of 331 fps through a CCD camera. Images were stored on a computer, and the contour of the droplet on the solid surface was processed by the Young–Laplace equation^62^ on a sample of 100 photographs between the third and sixth seconds of capture. For each image, the contact angle on the left and right sides of the droplet was determined, and the average value of the contact angle was calculated. Ten separate locations on the silicon wafer and five separate locations on each multilayer film were measured to ensure a representative value of the contact angle. The average value of the measured contact angles with its standard error of the mean was used to represent the wetting properties of the samples.

### 2.4 Atomic Force Microscopy (AFM)

The topography, surface roughness, and thickness of PAH/PAA films were determined by soft tapping mode atomic force microscopy (AFM) using a Multimode 8E AFM apparatus from Bruker. The used NCHV-A silicon probes (Bruker) were 117 μm in length and 33 μm in width with a resonance frequency of ∼320 kHz and a nominal spring constant of 40 N/m. The tip height was 10–15 μm, having a nominal radius of curvature of 8 nm. All of the AFM measurements were carried out in ambient air conditions. The temperature was (24 ± 2) °C and the relative humidity was between 30 and 50% during the measurements. All AFM scans were done on a 5 μm × 5 μm area with a scanning rate of 1 Hz and a picture resolution of 512 pixels × 512 pixels. After the data were processed in NanoScope 9.7, AFM images were corrected for tilt and bow using a second-order flattening and were analyzed in NanoScope Analysis 2.0 software to determine the local root-mean-square (RMS) roughness of LbL films. The AFM roughness parameters and appropriate standard errors of the mean reported here were calculated from all of the measurements, which included five local areas on each sample surface. Multilayer thickness was determined by gently removing a portion of the film from the substrate surface with a sharp tweezer and analyzing the cross sections of the scanned image. Details of the thickness measurements are described in the Supporting Information.

### 2.5 Ellipsometry

Thickness measurements for thin LbL films on the silicon substrate were made on an L116B-USB ellipsometer from Gaertner Scientific Corporation. The measurements were performed under ambient conditions using red He–Ne laser light with a wavelength of 632.8 nm at a fixed incident angle of 70° (close to the Brewster angle of the silicon/air interface, θ_B_ = 75.5°).^63^ For calculation of PEM thickness from the measured values of the amplitude ratio (*Ψ*) and change in phase (*Δ*), the commercial Gaertner Ellipsometric Measurement Program (Version 8.071) package was used. In the software, a three-box model with air as a continuum (*n* = 1.00),^64^ multilayer as a one-phase system with a refractive index of 1.55 that is independent of film composition and thickness, and Si wafer as a substrate was assumed.^65^ The Si/SiO*_x_* substrate was treated as a one-phase system, and before film thickness measurements, its average refractive index was determined by ellipsometric measurements on 10 different positions on each used Si plate. Multilayer thicknesses were determined at 10 different locations on each sample and are presented as an average (with standard error) of measurements for two individual films.

## 3 Results

### 3.1 Wetting Properties of the PAH/PAA Multilayer

**Figure 1:**
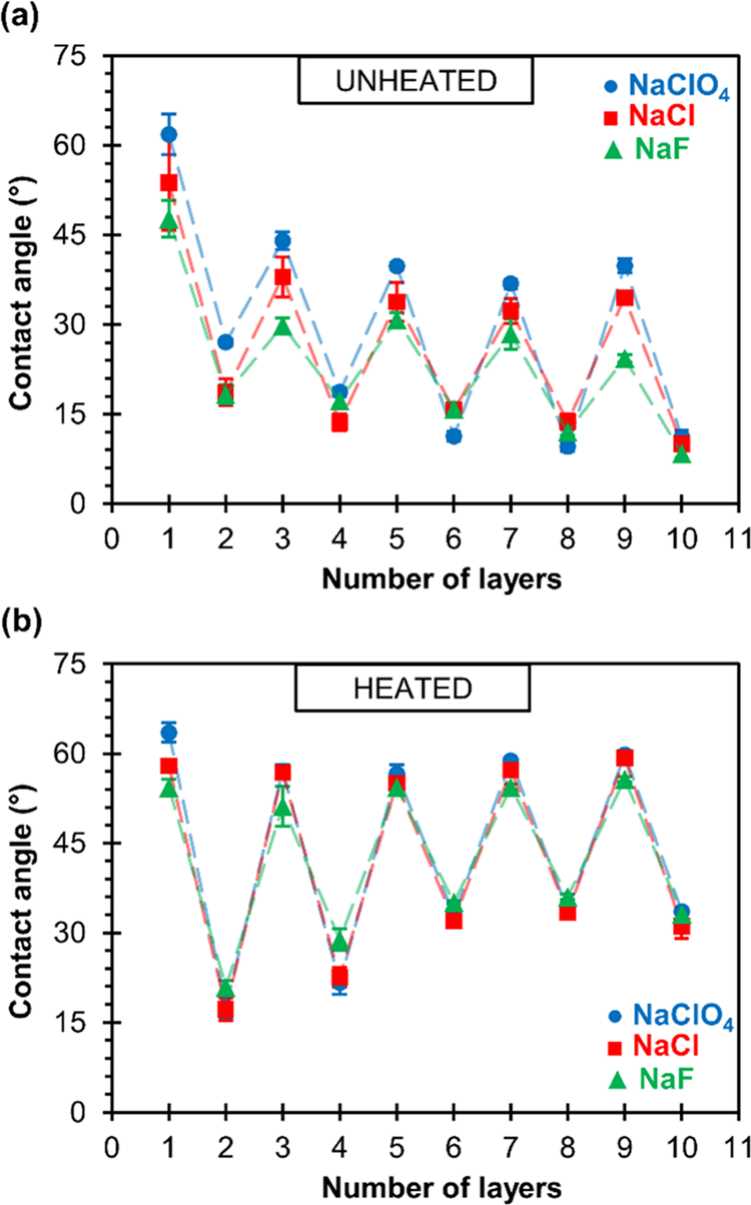
Water contact angles determined during buildup of PAH/PAA multilayers presented as a function of the number of layers. PEMs were prepared at pH = 7.0 from polyelectrolyte solutions (*c*_m_ = 0.01 M) containing different sodium salts (*c* = 0.10 M). Between each adsorption cycle, the samples were dried with (a) nitrogen and (b) nitrogen followed by heating at 60 °C for 30 min. Odd numbers represent films with PAH as the outermost layer, whereas even-numbered films have PAA as the outermost layer. Dashed lines have no physical meaning and were added as guides to the eye.

It is well known that in the solution counterions are associated to charged monomer units of polyelectrolytes. The conformation of such macromolecules will depend, among other factors, on the number of associated counterions. This claim holds for the behavior of polyelectrolytes both in a solution and on a surface.^66^ Therefore, if specific binding of counterions takes place, it could be expected that the conformation of polyelectrolytes on the surface would depend on the type of counterions. To determine how these ionic phenomena affect the surface wettability of PEMs, samples having five PAH/PAA bilayers were built up from solutions containing different salts and the contact angle was measured. Figure 1a displays the results of films fabricated by the dipping–rinsing–drying technique with PAH and PAA solutions containing 0.1 M NaF, NaCl, or NaClO_4_. To study the ion influence in more detail, PEMs were also prepared by heating the films after the drying step. Therefore, the first regime involved drying each layer of PEM in a stream of nitrogen, and the second regime was performed analogously to the first with heating of the sample for 30 min at 60 °C. The results of this additional heating step of PEMs are given in Figure 1b.

The contact angle of unheated and heated PAH/PAA multilayers shows the zig-zag dependance on the number of deposited polymer layers. This kind of pattern is typical for the polyelectrolyte type of LbL films and is known in the literature as the “odd–even effect”.^67−70^ We observed that less hydrophilic layers of PAH (θ ≈ 55°) and more hydrophilic layers of PAA (θ ≈ 15°) alternate on the surface. It is often the case that polycation layers are more hydrophobic than polyanion layers in the “odd–even effect”,^68−70^ but there are exceptions.^71^

As can be seen in Figure 1, the first layer of adsorbed PAH has a slightly higher contact angle (θ ≈ 60°) than the other layers closer to the multilayer/air interface. Furthermore, for the initial few layers of unheated films (Figure 1a), there is a trend of reducing the contact angle of PAH and PAA polyelectrolyte layers to more or less constant values. Note, however, that polyelectrolyte multilayers prepared by heating at 60 °C have an opposite trend (Figure 1b), i.e., if the terminating layer is a polyanion, then the contact angle increases from the initial 17° to the value of about 30° after the sixth layer. Unlike polyanions, when the terminating layer is a polycation, it has the same contact angle of ∼56° from the very first layer. Also, it should be noted that regardless of if PAH or PAA is the outermost layer, the contact angle is always greater (even 30°) for specimens that have been exposed to slightly elevated temperatures after normal drying in a stream of nitrogen.

Moreover, the results presented in Figure 1a show that the type of background salt used in the preparation of PEMs influences the contact angle of the individual PAH layer. In the absence of additional heating, the surface of the PAH layer is more hydrophilic when the multilayer is formed from NaCl than from NaClO_4_. Hydrophilicity is even more pronounced when NaF is the supporting electrolyte. On the contrary, such an anion-specific effect is not visible when polyanion is the terminating layer. Interestingly, the influence of counteranions on the contact angle is lost by heating of the multilayer (Figure 1b). The disappearance of the ion-specific effect is even easier to notice for the average contact angles of the PAH- and PAA-terminating multilayers prepared by these two procedures (Figure S1).

**Figure 2:**
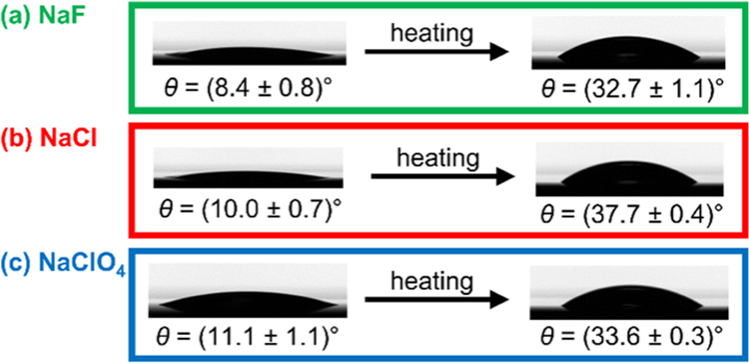
Advancing water contact angle (and standard error) of (PAH/PAA)_5_ multilayers measured before and after heating for 30 min at 60 °C. Multilayers were prepared without heat treatment at pH = 7.0 from polyelectrolyte solutions (*c*_m_ = 0.01 M) in a 0.10 M supporting electrolyte containing NaF (a), NaCl (b), and NaClO_4_ (c). Water droplets on multilayer surfaces are also presented.

To additionally clarify the reasons for the heating effect, the contact angles of silicon wafers previously coated with five PAH/PAA bilayers in a nonheating regime were measured before and after exposure to 60 °C for 30 min (Figure 2). Regardless of the supporting electrolyte used, after the heat treatment, the contact angle of the (PAH/PAA)_5_ multilayers increased from ∼10 to 33°. For comparison, the contact angles of (PAH/PAA)_5_ multilayers prepared in different salt mediums and exposed to heat treatment after each adsorption step are between 31 and 34° (Figure 1b). As the contact angles of (PAH/PAA)_5_ multilayers prepared by heating films in each adsorption step and by heating films after the preparation in the nonheating regime are almost the same independent of the background electrolyte used, we conclude that the surfaces of studied systems are in a similar state.

### 3.2 Surface Roughness of the PAH/PAA Multilayer

**Figure 3:**
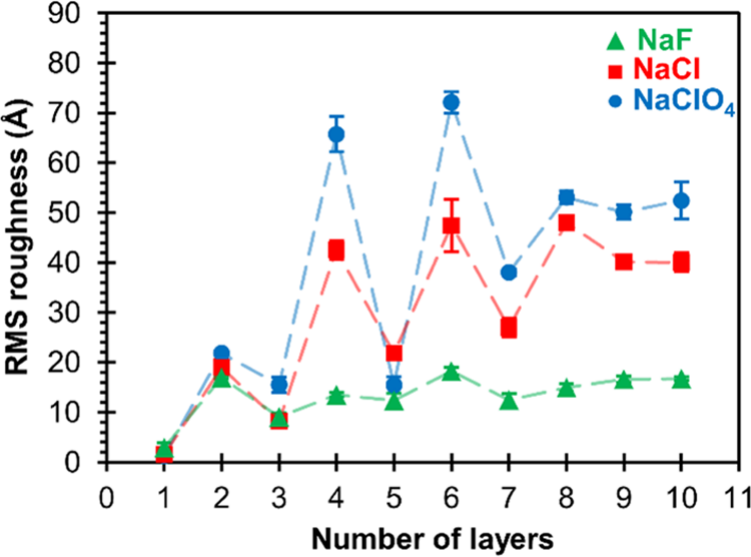
RMS surface roughness of the PAH/PAA multilayer as a function of the number of polyelectrolyte layers deposited on the surface of a silicon wafer from 0.01 M polyelectrolyte solutions at pH = 7.0 containing different sodium salts (*c* = 0.10 M). The samples were dried with nitrogen between each adsorption cycle, and no heat treatment was applied. Odd numbers represent films with PAH as the outermost layer, whereas even-numbered films have PAA as the outermost layer. Dashed lines have no physical meaning and were added as guides to the eye.

To investigate the changes in surface morphology upon adsorption of polyelectrolyte layers onto the Si substrate, AFM images of PAH/PAA multilayers were taken after each adsorption cycle (see Figures S2–S4). This allows even the study of the growth mechanism of the initial five PAH/PAA bilayers in NaF, NaCl, and NaClO_4_ environments. Here, we will consider the surface topography changes (deduced from the evolution of the surface roughness) during (PAH/PAA)_5_ multilayer buildup (Figure 3).

After cleaning silicon substrates with Piranha solution, we observed a smooth surface (Figure S5) with an RMS roughness of only (0.9 ± 0.1) Å. After the first PAH layer was formed, almost no coating could be seen on the surface regardless of the supporting electrolyte used (Figures S2–S4). As a result, the surface roughness was similar to the surface roughness of the substrate. Adsorption of the first PAA layer on the PAH monolayer produced a roughness increase and visible morphological changes at the surface. A grain-like topography was noticed for one PAH/PAA bilayer prepared in all three background salts. However, for PEMs prepared in the presence of NaF, this granular structure persisted up to the 10th layer and the roughness did not significantly change (Figure S2), and PEMs prepared in the presence of NaCl and NaClO_4_ exhibited different behaviors (Figures S3 and S4). In these cases, the surface topography alternately changed between the second and eighth layers from grain-like (even PAA layers) to blob-like (odd PAH layers) structures and then became more or less worm-like for both used salts. Subsequently, as seen in Figure 3, surface roughness changes in a zig-zag pattern as PAH and PAA layers alternately adsorb at the surface, becoming smoother after being coated with PAH and rougher after being coated with PAA.

It is important to note that the choice of background salt affected the topography and the roughness significantly. One can see the difference in roughness between the PEMs prepared in the presence of NaF, NaCl, and NaClO_4_ (Figure 3). For the same number of layers, PEMs prepared from the NaClO_4_ solution show a larger surface roughness than PEMs prepared from NaCl and especially NaF solution. For instance, the local RMS roughness of the (PAH/PAA)_5_ film prepared from NaClO_4_ is around 3 times higher than the roughness of the same film prepared from the NaF solution, which is comparable to the results observed for the PAH/PSS assembly.^38^

**Figure 4:**
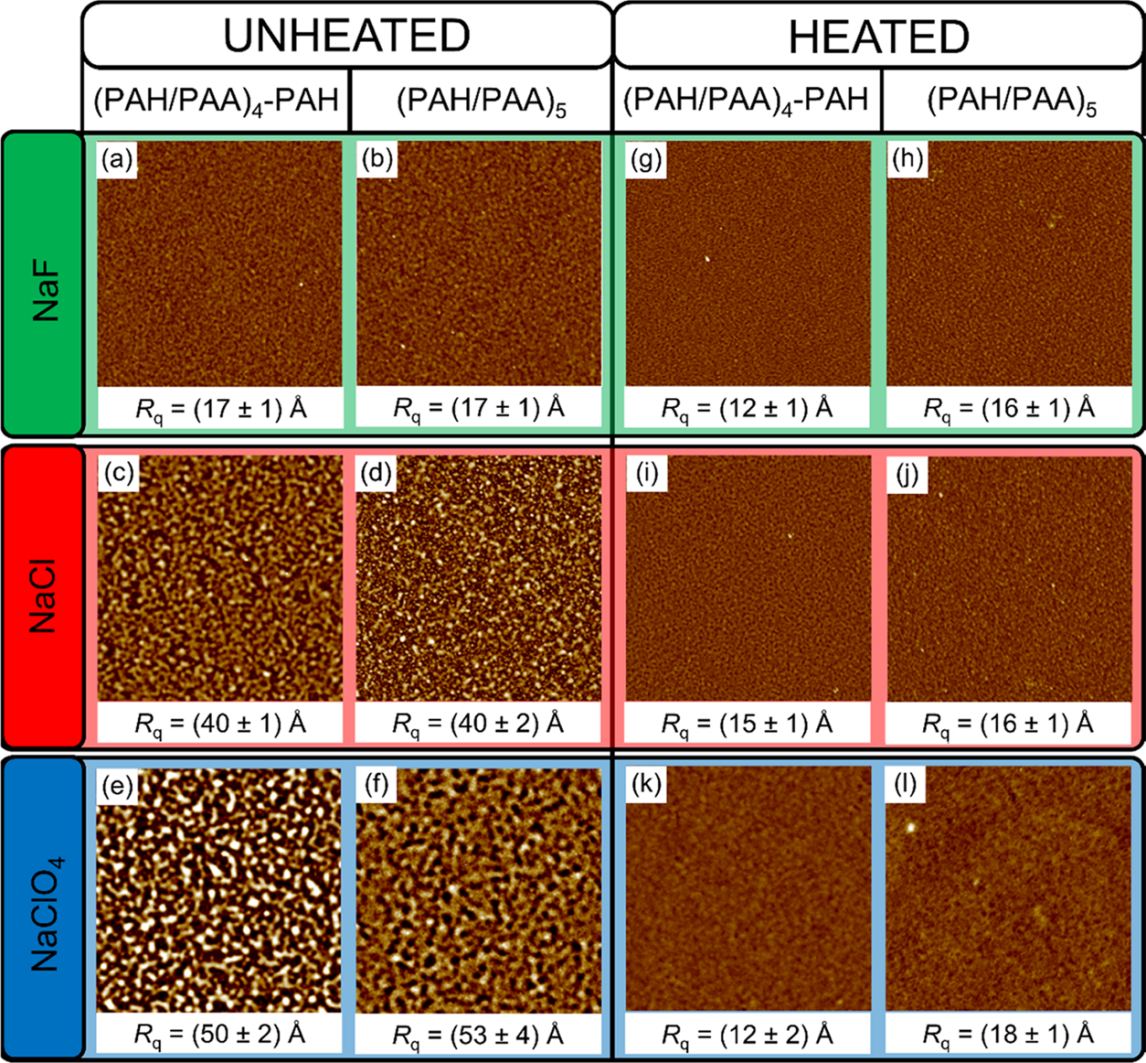
Surface topography (AFM images) of unheated (a–f) and heated (g–l) PAH/PAA films with 9 and 10 layers prepared from polyelectrolyte solutions containing 0.1 M NaF (a, b, g, h), NaCl (c, d, i, j), and NaClO_4_ (e, f, k, l). The images have a scan size of 5 × 5 μm^2^, and the *z*-scale is set to 30 nm. The corresponding RMS roughness values are shown below each image.

As in the case of surface wettability, heat treatment of films between each adsorption cycle dramatically affected the surface morphology and roughness. Figure 4 shows AFM images of unheated and heated multilayers with 9 (PAH-terminated) and 10 (PAA-terminated) layers prepared in F^–^, Cl^–^, and ClO_4_^–^-containing medium.

From Figure 4, it can be observed that the surfaces of the heated samples are smoother than the unheated ones regardless of whether the terminating layer is PAH or PAA. The surface smoothing is especially pronounced for PEMs prepared in NaCl and NaClO_4_ as supporting electrolytes. For example, RMS roughness parameters of unheated and heated (PAH/PAA)_4_-PAH films prepared in NaClO_4_ are 50 and 12 Å, respectively. Also, it is interesting to compare the surface roughness of unheated and heated films prepared in different background electrolytes. Whereas for unheated PAH/PAA multilayers, RMS roughness increases in the order F^–^ < Cl^–^ < ClO_4_^–^, for heated PAH/PAA multilayers, such ion specificity is suppressed. Regardless of the background salt used, the local roughness of heated multilayers is about 15 Å (Figure 4). Apparently, the heating caused structural modifications on the surface of the multilayers, which resulted in a change in the film’s surface roughness. The question is whether these changes also affected the structure of the film’s interior. If so, then that should be reflected in the thickness of the film itself. Therefore, we performed ellipsometric and additional AFM measurements, whose results are presented in the following chapter.

### 3.3 Thickness of the PAH/PAA Multilayer

**Figure 5:**
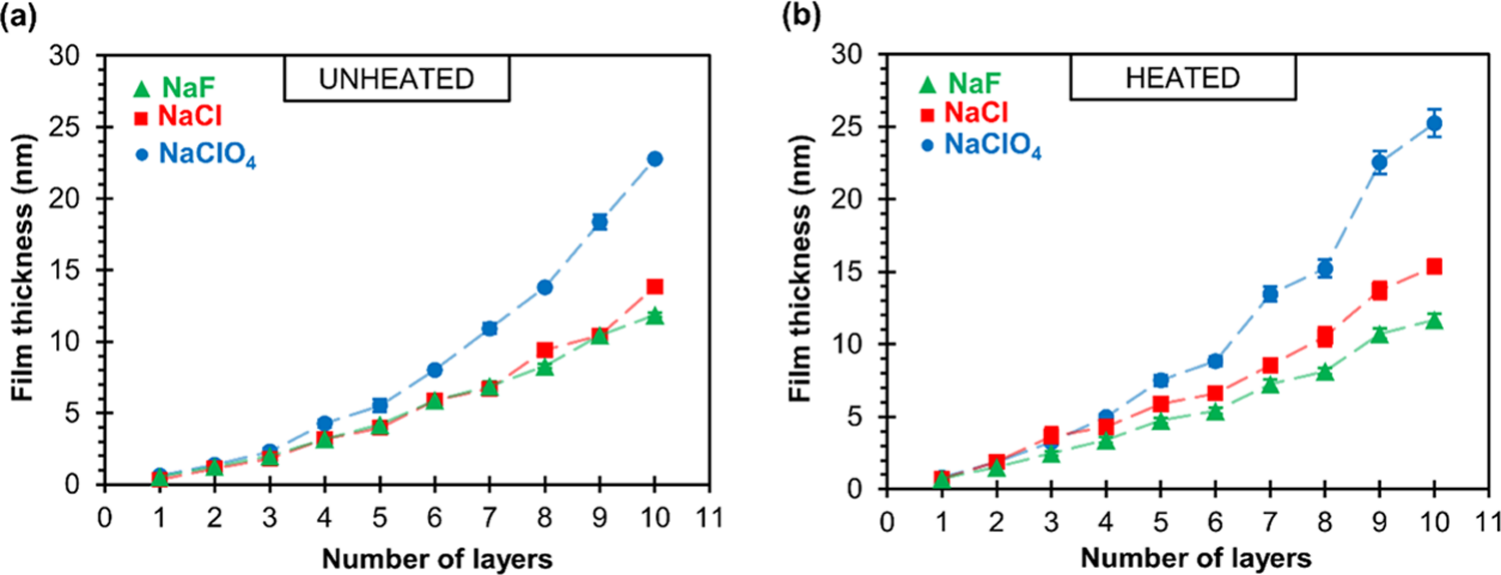
Ellipsometric thickness of unheated (a) and heated (b) PAH/PAA multilayers as a function of the numbers of deposited polyelectrolyte layers on the Si substrate. Multilayers were assembled from 0.01 M polyelectrolyte solutions with 0.1 M sodium salt of the examined anions. Dashed lines were added as guides to the eye.

The thickness of PAH/PAA multilayers was measured using both ellipsometry and AFM. Figure 5a shows the film thickness obtained ellipsometrically during the growth of unheated PAH/PAA multilayers. In the case of all three examined salts, the film thickness exponentially increases with the number of deposited polyelectrolyte layers. This exponential growth of the film is the most pronounced for multilayers prepared in NaClO_4_. The difference in thickness between unheated multilayers prepared in the presence of NaCl and NaF is rather small and is only noticeable in films with five bilayers. However, after heating (Figure 5b), the difference in the thickness of the multilayers prepared with NaF and NaCl increased and the film growth in the series of fluorides, chlorides, and perchlorates is more apparent. Although the heated multilayers are only a nanometer to two thicker than unheated films, it seems that heat treatment of PAH/PAA films during the LbL process further accentuates ion specificity with respect to thickness. This result suggests that the internal structure of the PAH/PAA multilayer did not change significantly upon heating. Also, it should be noted that the exponential type of film growth remains.

**Figure 6:**
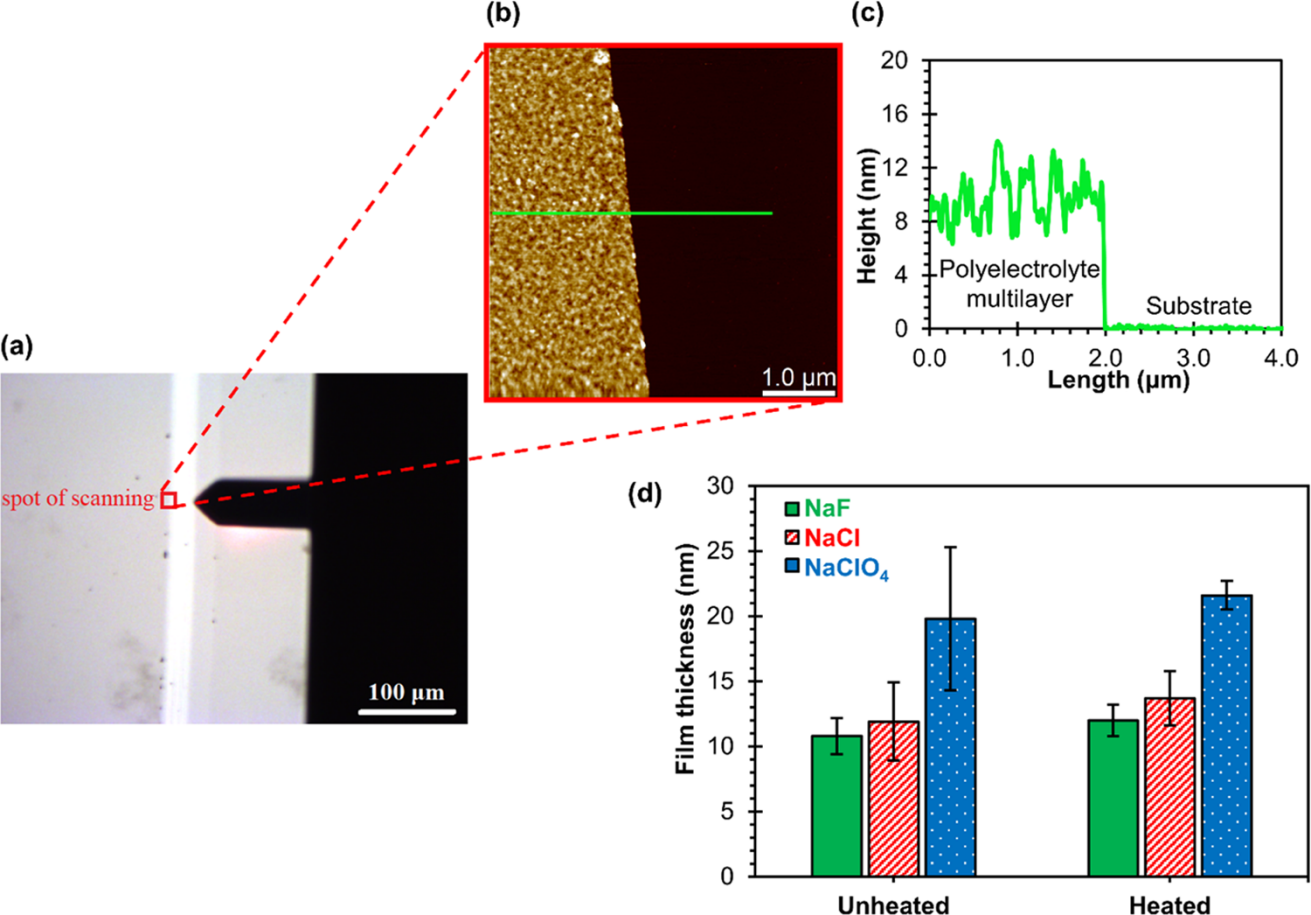
Area where the unheated (PAH/PAA)_5_ multilayer was removed from the surface of the Si substrate, recorded by (a) a digital optical microscope and (b) an atomic force microscope. The PAH/PAA film was assembled at pH = 7.0 from 0.01 M polyelectrolyte solutions with 0.1 M NaF as a background electrolyte. (c) Height profile crosses the green line designated in the AFM image. (d) Total AFM thickness of unheated and heated (PAH/PAA)_5_ multilayers deposited on the Si surface in 0.1 M sodium salt of the corresponding anions.

To determine the thickness of PAH/PAA multilayers using AFM, the multilayer was partially removed from the substrate surface with sharp-tipped tweezers, as described in the Supporting Information. The surface was then imaged in the scratched area with a digital optical microscope and AFM. Representative microscopic images of the area where the multilayer was removed from the surface of the Si substrate are shown in Figure 6a,b. In Figure 6a, the white line represents part of the surface from which the polyelectrolyte film was removed, and Figure 6b shows the AFM image of the area along the very edge of this white line. From the AFM image presented in Figure 6b, a flat surface of the substrate “connected” to the rougher surface of the multilayer could be observed. By the detailed analysis of the height profiles (example presented in Figure 6c), the thickness of unheated and heated (PAH/PAA)_5_ multilayers prepared in different supporting electrolytes was determined. The results are presented in Figure 6d. Again, the films prepared in NaF are the thinnest, the films prepared in NaCl are of medium thickness, and the thickest films are prepared in NaClO_4_. As in the case of ellipsometric measurements, this influence of anions on the film thickness is somewhat more pronounced for heated samples. Also, the average thickness of heated multilayers is slightly higher than the thickness of unheated ones.

The AFM thickness determination for unheated and heated (PAH/PAA)_5_ multilayers (Figure 6d) gives the same order of thickness for the supporting anion as measured by ellipsometry (Figure 5). However, it is noteworthy that for unheated multilayers, thickness values determined by AFM are 10–15% smaller compared to the values determined ellipsometrically (Table S1). Such a result raises a question about the refractive index used for fitting the ellipsometric results (*n* = 1.55). On the other hand, the AFM measures the actual thickness of a film ignoring the possible material defects inside the film, whereas in ellipsometry, film homogeneity and a high degree of surface smoothness are required for reliable results.

Upon closer analysis, one can find, from the value of error bars (Figure 6d), that the fluctuations in thickness are smaller for heated than unheated films, meaning a smaller surface roughness. This observation is in good agreement with the results of roughness experiments shown in the previous section (Figure 4). Furthermore, the value of error bars for unheated samples follows the increase in RMS surface roughness in the NaF, NaCl, and NaClO_4_ trend.

## 4 Discussion

To explain our results more systematically, this section is divided into two parts. In the first part, we describe the anion dependency of PAH/PAA multilayer properties, whereas in the second part, the observed influence of heating on PAH/PAA multilayer properties is discussed.

### 4.1 Anion-Specific Effect

In this study, we used silicon wafer as a substrate for PAH/PAA multilayer formation in NaF, NaCl, and NaClO_4_ electrolyte mediums. The biggest problem in the assembly of weak polyelectrolyte pairs is finding the appropriate pH for PEM buildup. Here, the pH value of 7.0 was chosen for multilayer deposition because at this condition both polyelectrolytes have maximal charge density.^60^ As at pH = 7.0, the surface of the Si wafer is highly negatively charged (ζ-potential ≈ −50 mV),^72^ the first adsorbed polyelectrolyte layer was always PAH. After the first PAH layer was formed, almost no coating could be seen on the surface and the roughness did not change (Figure 3). However, the contact angle significantly increased from 25 to around 55° (Figure 1). For comparison, Fujita and Shiratori^73^ observed the same result for PAH adsorption on the Si wafer surface at pH = 7.5. In addition to AFM and contact angle measurements, they used a quartz crystal microbalance and X-ray photoelectron spectroscopy (XPS) to further examine the adsorption of PAH. On the basis of the obtained results, the authors concluded that a coating was formed. Therefore, we can assume that a thin compact PAH layer fully covered the Si surface due to the strong electrostatic attraction between the negatively charged substrate and the positively charged PAH molecules.

**Figure 7:**
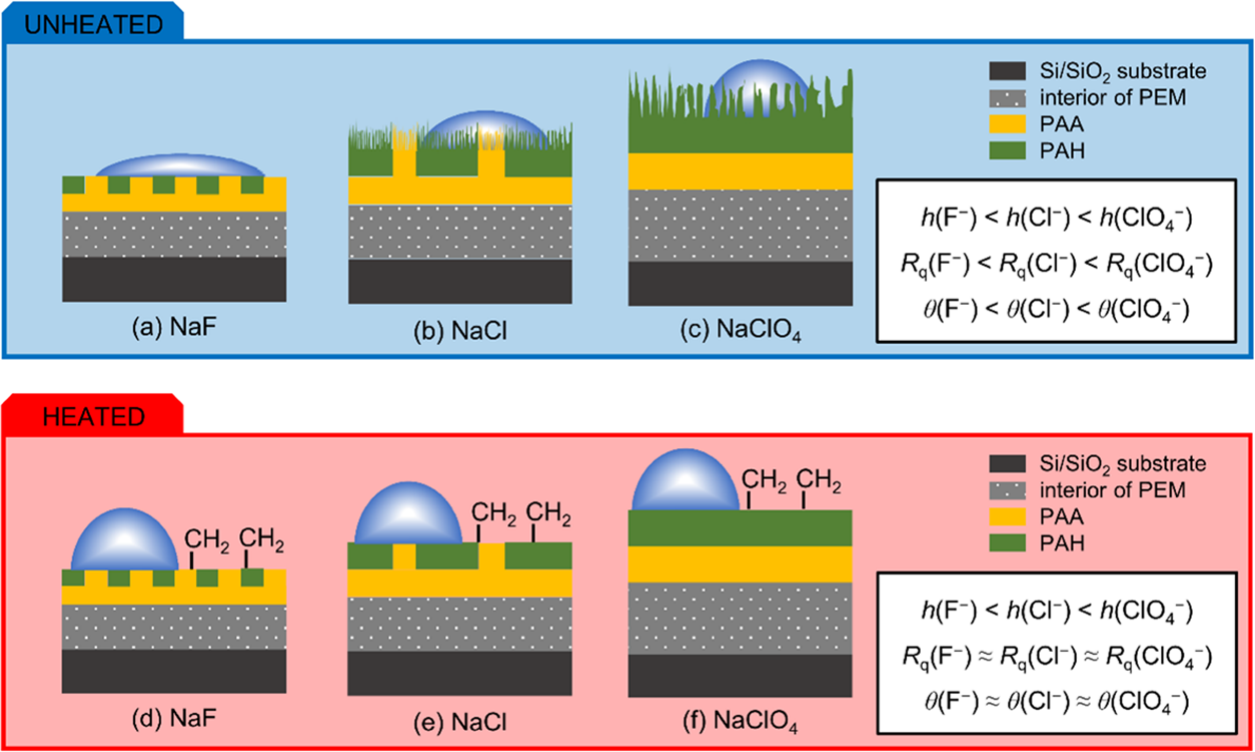
Models of unheated (a–c) and heated (d–f) PAH/PAA multilayers prepared in different supporting electrolytes. The insets show the relationship among film thickness (*h*), surface roughness (*R*_q_), and contact angle (θ) of the multilayers prepared in different background salts.

As additional layers of PAA and PAH were successively deposited on the silicon surface, the film thickness and roughness increased (Figures 3 and 5). The thickest multilayers with the highest surface roughness were formed in NaClO_4_, while multilayers prepared in NaF were the thinnest and had the smoothest surface. LbL films prepared in NaCl were of medium thickness and roughness (Figure 7). Undoubtedly, this ranking of anions (F^–^ < Cl^–^ < ClO_4_^–^) is in accordance with the Hofmeister series.^49^ As observed for polyelectrolyte complexes, PAH monomeric units prefer to associate larger oxoanions with smaller hydration shells (e.g., ClO_4_^–^), as opposed to the relatively small anions of halogen elements (e.g., Cl^–^).^74^ That means that ClO_4_^–^ ions have a greater ability than Cl^–^ or F^–^ ions to screen the free charges of PAH molecules in solution. As a result, electrostatic repulsions between charged polymer segments of PAH will be weaker in ClO_4_^–^ solution than in Cl^–^ or F^–^ solutions, and PAH chains will adopt a more globular form in NaClO_4_ solution. This leads to polyelectrolyte deposition on the substrate surface in loopier conformations. Therefore, a higher amount of polycation is needed in the surface charge compensation for the NaClO_4_ case, causing thicker films with more pronounced surface roughness. On the contrary, in NaCl and NaF solutions, PAH chains will be in a more linear form and the films will be thinner and smoother.

In addition to the influence of the type of background salt on the thickness and roughness of the PAH/PAA multilayer, it was also shown that the wettability of the film surface depends on the supporting anion used. As with thickness and roughness, it was found that the hydrophobicity of the surface increases in the series F^–^ < Cl^–^ < ClO_4_^–^ when the terminating layer is PAH (Figure 7). To discuss these results, it is important to consider the relevant factors that affect the contact angle such as the molecular structure of the polyelectrolyte in the terminating layer, surface roughness, and the level of interlayer interpenetration.

Many studies^67−69^ have shown that the polyelectrolytes located inside the multilayer do not have any impact on the interactions of water molecules with the surface and that the surface wettability of sequentially adsorbed polyelectrolyte layers is controlled primarily by the terminating layer. In that manner, whether the polyelectrolyte layer on the surface will be hydrophobic or hydrophilic depends on the structure of the macromolecule. In our case, both the polycation and the polyanion are hydrophilic due to polar amino and carboxylic groups. On the basis of the molecular structure, it is difficult to conclude which polymer is more hydrophilic, but one can assume that PAA makes stronger interactions with water molecules due to the greater possibility of making hydrogen bonds, as recently suggested by molecular dynamic simulations.^75^ The mentioned structural properties of used polyelectrolytes could be the reason behind the observed odd–even effect in the hydrophilic region of contact angles (Figure 1), but they cannot explain the observed ion-specific effect.

Even the PAH monolayers assembled on the substrate surface in different salt conditions show a difference in surface wettability. Potential explanation of this effect could lie in the higher proportion of amino groups screened by anions, which results in a higher hydrophobicity due to weakening of polyelectrolyte–water interactions. Therefore, the PAH monolayer has the highest hydrophobicity with perchlorates and the lowest with fluorides. Such surface wettability behavior is also propagated to films with a higher number of layers. However, the values of contact angles are lower compared to the values for the PAH monolayer (Figure 1a). This first layer effect reflects different deposition properties expected when PAH is deposited directly onto the hydrophilic substrate and, perhaps more importantly, the fact that it is not influenced by the presence of an underlying layer of PAA. Nevertheless, Wang et al.^76^ have demonstrated how counterions present at the surface of PEM could be utilized for the modulation of surface wettability *via* an ion-exchange mechanism. In their study, an as-made PDADMAC/PSS film was dipped in aqueous solutions of different anions, and the observed water contact angle of the surface varied from about 10 to 120°. These results indicate that the variation in the surface wettability of the PAH/PAA multilayer reported here (Figure 1) could be partially attributed to the hydration capability of the counterions present at the film surface.

Except for the structural parameters, another important factor affecting the wetting properties of a multilayer is its roughness. If the multilayer is nonporous and has a homogeneous surface, the contact angle will be influenced by the roughness of its surface. According to Wenzel’s theory,^77^ a contact angle of more than 90° is increased by the roughening of the surface, and the one of less than 90° is reduced. Within the framework of Wenzel’s equation, surface roughness is expressed by Wenzel’s factor, which is defined by the ratio of the actual and geometric surface area. Detailed analysis of our AFM images revealed that the highest Wenzel’s factors for PAH-terminated multilayers prepared in NaF and NaClO_4_ solutions are 1.01 and 1.03, respectively. This order of values indicates that both surfaces are very flat and the difference between them is not enough to explain the variations in contact angles of LbL films prepared in different salts.

The final parameter that affects the wetting of a multilayer surface is interlayer interpenetration, i.e., if the segments of the previously adsorbed layer have penetrated the surface of the outermost adsorbed layer, the wettability of the multilayer film will change. As Rubner and co-workers explained,^68^ if the surface layer of PEM is made of a hydrophobic polyelectrolyte, then decreasing the thickness of that layer or increasing the thickness of the previously adsorbed hydrophilic layer decreases the contact angle, suggesting that the hydrophilic segments of the underlying layer penetrate the surface layer. In general, whenever a thin layer is deposited onto a thicker layer, a large number of chain segments from the sublayer penetrates the outermost layer and that way affects the measured contact angle of the film. As LbL films made in the NaF solution have the thinnest layers, it could be expected that they have a high level of interlayer interpenetration, which results in the lowest contact angles when PAH is the terminating layer. In the case of films prepared in NaCl and especially NaClO_4_, polyelectrolyte layers are thicker and thus more discrete with a low level of interlayer interpenetration. Consequently, these multilayers will have higher contact angles when PAH is the outermost layer (Figure 7).

As PAA layers are generally thicker than PAH layers (Figure S6), the interpenetration of polyelectrolytes between layers will be more pronounced for PAH-terminated multilayers. Furthermore, anions do not have a direct effect on the negatively charged PAA functional groups, so it is expected that the contact angle of PAA surface layers will be very similar regardless of the sodium salt in which the multilayers were prepared. Of course, the contact angle of the PAA surface may vary slightly with the background sodium salt used, but this is due to the small indirect influence of the adjacent PAH layer, which is, as mentioned before, subject to anionic screening.

It is important to point out that the choice of the supporting electrolyte for the LbL assembly also affects the composition of a PEM. Within the multilayer, a polyelectrolyte repeating unit can be compensated either by an oppositely charged repeating unit (intrinsic site) or by a counterion (extrinsic site).^78^ The extrinsic charge is present in a film due to the nonstoichiometric composition of polyelectrolytes within the multilayer. The fraction of extrinsic sites in PEM markedly depends on the salt concentration but also on the salt type present in the dipping solution.^79^ The type of salt is the most important during the adsorption process because the polyelectrolyte/counterion binding constant is the one that exhibits ion specificity, i.e., different polyelectrolytes and different salt ions are expected to interact with different strengths. Recently, it was reported that PAH chains prefer the binding of weakly hydrated oxyanions such as perchlorates over strongly hydrated halide anions (e.g., F^–^ and Cl^–^).^74^ Therefore, it is to be expected that the PAH/PAA multilayer prepared in the presence of NaClO_4_ will have a higher ion content than the same film prepared in the presence of NaCl or NaF. In addition to polymers and ions, PEMs, in most cases, contain a significant amount of water.^80^ Increasing the salt content of PEM usually results in an increase of the overall water content as the salt ions carry water into the assembly.^30^ A rationalization of the difference between the water content of PEMs prepared in three examined salts may be reached by considering ion hydration and efficiency of doping the PAH/PAA multilayer with ions. As explained by Schlenoff and co-workers,^79^ the less hydrated the ion is in the solution, the more effective it is at hydrating the polyelectrolyte multilayer. Such a trend is a consequence of the significantly better doping of PEM by less hydrated ions. According to this concept, the amount of water present in as-made PAH/PAA multilayers should increase in the same order as the concentration of extrinsic sites (F^–^ < Cl^–^ < ClO_4_^–^). In total, both ion and water contents of the PAH/PAA multilayer increase on decreasing the ion hydration.

### 4.2 Heating Effect

Here, for the first time, we demonstrate how the heating of PAH/PAA multilayers for 30 min at 60 °C can significantly suppress the impact of anions on some film properties. For example, heat treatment of PAH/PAA multilayers resulted in an increase of their thickness and hydrophobicity, while the surface roughness decreased (Figure 7). More importantly, the contact angle and surface roughness of the PEMs were almost the same after the heat treatment independent of the background electrolyte used (Figure 7), leading to the conclusion that the studied systems are in a similar state of surface structure.

One possible explanation for the observed results could be the glass transition of PEMs upon heating. However, the irreversible nature of observed changes in the properties of annealed films excludes this possibility. Moreover, the Lutkenhaus group has demonstrated that, unlike wet,^81^ dry PAH/PAA multilayers do not exhibit glass-transition temperature (*T*_g_) and instead undergo anhydride formation between carboxyl groups of PAA, and amidation of −NH_3_^+^ and −COO^–^ groups.^82,83^ Furthermore, the authors have reported that intra- and inter-anhydride PAA bonds are formed at temperatures between 80 and 120 °C and amidation takes place at even higher temperatures (>200 °C). In our work, PAH/PAA films were heated only up to 60 °C so that formation of anhydride and amide bonds in films could also be excluded.

Another explanation for the observed heating effect could be water evaporation from PEMs. As explained by von Klitzing and co-workers,^84,85^ PEM consists of two types of water, namely, void and swelling water. The former only fills the voids that are formed during the multilayer preparation process, while the latter directly contributes to the change in multilayer thickness. As the multilayer adsorbs water at higher relative humidities, the amount of swelling water and the thickness of the film increase. On the other hand, lowering the relative humidity will produce thinner films. Taking these facts into account, one would expect that after the heating process PAH/PAA multilayers would be thinner as they lose swelling water, but our experiments indicated that films are slightly thicker. Moreover, we ascertained that the heat treatment drastically changes the surface morphology of the films. This could not be only the consequence of the deswelling process.

Therefore, in our opinion, the main origin of the observed heating effect is interdiffusion and reorientation of polyelectrolyte molecules present at the multilayer–air interface. The diffusion process within the LbL assemblies was extensively studied for the past two decades by the Schlenoff group.^86−88^ They concluded that there are at least three types of diffusing species within PEM: ions, polymer chains, and extrinsic polyelectrolyte–counterion sites. We are convinced that in our experiments, the amount of energy that was brought to multilayer systems by heating was enough to overcome the energy barriers needed for the migration of all three diffusing species. Although all of these species migrate in PEM during annealing, the rate of their transport in the film is a few orders of magnitude different. As reported by Fares and Schlenoff,^87^ ion diffusion is the fastest of all species, the diffusion of extrinsic sites is markedly slower, and the migration of polyelectrolytes themselves is the slowest.

Because polymer diffusion demands massive transport of a material, it is usually too slow to allow access of the polymer to an entire film (even at higher temperatures).^88^ As a result, this migration of macroion chains takes place only in the interface region and thus causes morphological changes in the surface that were directly visualized here by AFM imaging (Figure 4). In another AFM study, Ghostine et al.^89^ showed that exposing PEMs to a salt solution (“salt annealing”) frees polyelectrolyte segments and allows polymer interdiffusion, leading to the smoothing of the surface. Similarly, thermal annealing performed here enhances polymer motions and liquefies the film surface by partial reorganization of the bonds between the oppositely charged polyions. As the migration of polymer chains tends to minimize the surface energy of the initially metastable PEM structure,^66,90^ the material in the “peaks” diffuses into the “valleys”. Consequently, heated multilayers have lower surface roughness than unheated ones. Moreover, as only the top layers are affected by migration and this process happens in a dry state, heated films are only slightly thinner than unheated ones and the ion specificity is preserved in terms of thickness. On the contrary, the effect of salt on PEM roughness is lost after the heating because the surface of annealed films is in its configuration of the lowest energy.

To explain the increased hydrophobicity and suppression of contact angle ion-dependency observed for heated PEMs, we suggest that heat treatment transforms some of the hydrophilic moieties on the multilayer surface to −CH_2_-rich ones. The support for our hypothesis has been presented in a recent study by Gustafsson et al.^91^ The authors used the interface-sensitive vibrational sum frequency spectroscopy (VSFS) technique to investigate heat-induced molecular rearrangements at the PAH/PAA multilayer surface and showed an increased CH_2_ signal after heat treatment, indicating reorientation of chemical constituents in the outermost layers. This transformation causes a rise of the contact angle to similar values regardless of the used background salt because −CH_2_ groups are not affected by anion association (Figure 7). Also, the interpenetration of polyelectrolytes between layers will not have an influence on surface wettability because segments of both polyelectrolytes would be oriented so that −CH_2_ groups are exposed on the surface. This is also supported by contact angle measurements that showed no change in the contact angle after 15 h or additional heating of LbL films prepared with heating in all three examined salts (Figure S7). This means that the surface is stable and that the fraction of -CH_2_ groups on the surface is maximal for these conditions.

Finally, we would like to emphasize the important role of ions and water in the thermal annealing of PEMs. Commonly, ions and water are considered to be plasticizers of polyelectrolyte assemblies.^92,93^ The presence of salt ions in PEMs provides additional free volume for chain motion and weakens PAH–PAA ion pairing due to electrostatic screening, which contributes toward plasticization of the film. Similar to ions, water molecules influence polymer motions by increasing the effective volume of the polyelectrolyte multilayer and decreasing the friction between polymer chains. As elaborated earlier, both ion and water contents of the PAH/PAA multilayer depend on the type of salt present in the assembly bath. Among NaF, NaCl, and NaClO_4_, the PAH/PAA multilayer prepared in the presence of NaClO_4_ has the highest amount of extrinsic sites and water. This makes the chaotropic ClO_4_^–^ ion a better plasticizer than the Cl^–^ ion or the cosmotropic F^–^ ion. However, one should keep in mind that the amount of water present in PEM decreases with rising temperature if the film is exposed to ambient conditions.^80^ The reduction of water content in PEM limits the movements of the polymer chains and potentially affects the plasticization ability of the ions.

## 5 Conclusions

The effect of a supporting anion on properties of PAH/PAA LbL films was studied using ellipsometry, AFM, and tensiometry. We have observed that in thin dry multilayers of these weak polyelectrolytes the thickness and surface roughness follow the position of the anion in the Hofmeister series. This is in line with previous reports for strong–strong and weak–strong polyelectrolyte assemblies.^28,38^ Furthermore, we found that the surface wettability of the PAH/PAA multilayer also depends on the anion used in the deposition process when the polycation is the outermost layer. Observed ion-specific effects can be satisfactorily explained by the charge screening of the polyelectrolyte. Chaotropic anions (e.g., ClO_4_^–^) strongly screen PAH chain segments, inducing the deposition of polyelectrolytes in a loopy form onto a surface, yielding a thick layer with a high surface roughness. The assembly of relatively thick PAH layers on the polyanion surface results in a low level of polycation/polyanion interpenetration, leading to a small percentage of hydrophilic PAA chain segment on the PAH-layer surface. On the contrary, the cosmotropic anions such as F^–^ do not screen polyelectrolyte charges with the same strength, allowing the polyelectrolyte to deposit in a more planar conformation. Thus, individually deposited layers in the multilayer system are thin, smooth-faced, highly interpenetrated, and therefore more hydrophilic.

Additionally, for the first time, we have shown that thermal annealing of PAH/PAA multilayers between each polyelectrolyte adsorption step at 60 °C can completely suppress the influence of background anions on film roughness and wettability. It seems that the heat treatment induces changes at the polymer–air interface in the sense of reorientation and migration of polymer chains. These conclusions are well supported by VSF spectroscopy.^91^ Our future work will encompass an investigation of the influence of supporting cations on properties of PAH/PAA multilayers and suppression of this influence by the heating of the film. These results are expected to complement studies on the ion-specific and heating effects currently being explored in our laboratory.

## Supporting Information Available

Additional experimental details about film thickness measurements by AFM, average contact angle of PAH- and PAA-terminated multilayers, AFM images of PAH/PAA multilayers in a layer-by-layer manner, AFM image of the silicon substrate surface, thickness of each PAH and PAA layer during the multilayer buildup, contact angle of heated PAH/PAA films after 15 h and additional heating, and comparison of film thickness determined by an ellipsometer and AFM (PDF)

## References

[ref1] Decher G.; Hong J. Buildup of Ultrathin Multilayer Films by a Self-Assembly Process, 1 Consecutive Adsorption of Anionic and Cationic Bipolar Amphiphiles on Charged Surfaces. Makromol. Chem., Macromol. Symp. 1991, 46, 321–327. 10.1002/masy.19910460145.

[ref2] Decher G.; Hong J. D.; Schmitt J. Buildup of Ultrathin Multilayer Films by a Self-Assembly Process: III. Consecutively Alternating Adsorption of Anionic and Cationic Polyelectrolytes on Charged Surfaces. Thin Solid Films 1992, 210–211, 831–835. 10.1016/0040-6090(92)90417-A.

[ref3] de Grooth J.; Oborný R.; Potreck J.; Nijmeijer K.; de Vos W. M. The Role of Ionic Strength and Odd–Even Effects on the Properties of Polyelectrolyte Multilayer Nanofiltration Membranes. J. Membr. Sci. 2015, 475, 311–319. 10.1016/j.memsci.2014.10.044.

[ref4] Ilyas S.; Abtahi S. M.; Akkilic N.; Roesink H. D. W.; de Vos W. M. Weak Polyelectrolyte Multilayers as Tunable Separation Layers for Micro-Pollutant Removal by Hollow Fiber Nanofiltration Membranes. J. Membr. Sci. 2017, 537, 220–228. 10.1016/j.memsci.2017.05.027.

[ref5] Durmaz E. N.; Sahin S.; Virga E.; de Beer S.; de Smet L. C. P. M.; de Vos W. M. Polyelectrolytes as Building Blocks for Next-Generation Membranes with Advanced Functionalities. ACS Appl. Polym. Mater. 2021, 3, 4347–4374. 10.1021/acsapm.1c00654. 34541543 PMC8438666

[ref6] Zhai L.; Cebeci F. C.; Cohen R. E.; Rubner M. F. Stable Superhydrophobic Coatings from Polyelectrolyte Multilayers. Nano Lett. 2004, 4, 1349–1353. 10.1021/nl049463j.

[ref7] Zhai L.; Berg M. C.; Cebeci F. Ç.; Kim Y.; Milwid J. M.; Rubner M. F.; Cohen R. E. Patterned Superhydrophobic Surfaces: Toward a Synthetic Mimic of the Namib Desert Beetle. Nano Lett. 2006, 6, 1213–1217. 10.1021/nl060644q. 16771582

[ref8] Cebeci F. Ç.; Wu Z.; Zhai L.; Cohen R. E.; Rubner M. F. Nanoporosity-Driven Superhydrophilicity: A Means to Create Multifunctional Antifogging Coatings. Langmuir 2006, 22, 2856–2862. 10.1021/la053182p. 16519495

[ref9] Chung A. J.; Rubner M. F. Methods of Loading and Releasing Low Molecular Weight Cationic Molecules in Weak Polyelectrolyte Multilayer Films. Langmuir 2002, 18, 1176–1183. 10.1021/la010873m.

[ref10] Granicka L. H. Nanoencapsulation of Cells Within Multilayer Shells for Biomedical Applications. J. Nanosci. Nanotechnol. 2014, 14, 705–716. 10.1166/jnn.2014.9106. 24730291

[ref11] Yu W.; Chen Y.; Mao Z. Hollow Polyelectrolyte Microcapsules as Advanced Drug Delivery Carriers. J. Nanosci. Nanotechnol. 2016, 16, 5435–5446. 10.1166/jnn.2016.11748. 27427582

[ref12] Sharma V.; Sundaramurthy A. Multilayer Capsules Made of Weak Polyelectrolytes: A Review on the Preparation, Functionalization and Applications in Drug Delivery. Beilstein J. Nanotechnol. 2020, 11, 508–532. 10.3762/bjnano.11.41. 32274289 PMC7113543

[ref13] Bohinc K.; Kukić L.; Štukelj R.; Zore A.; Abram A.; Klačić T.; Kovačević D. Bacterial Adhesion Capacity of Uropathogenic Escherichia Coli to Polyelectrolyte Multilayer Coated Urinary Catheter Surface. Coatings 2021, 11, 630 10.3390/coatings11060630.

[ref14] Shim B. S.; Chen W.; Doty C.; Xu C.; Kotov N. A. Smart Electronic Yarns and Wearable Fabrics for Human Biomonitoring Made by Carbon Nanotube Coating with Polyelectrolytes. Nano Lett. 2008, 8, 4151–4157. 10.1021/nl801495p. 19367926

[ref15] Loh K. J.; Kim J.; Lynch J. P.; Kam N. W. S.; Kotov N. A. Multifunctional Layer-by-Layer Carbon Nanotube-Polyelectrolyte Thin Films for Strain and Corrosion Sensing. Smart Mater. Struct. 2007, 16, 429–438. 10.1088/0964-1726/16/2/022.

[ref16] Shiratori S. S.; Rubner M. F. PH-Dependent Thickness Behavior of Sequentially Adsorbed Layers of Weak Polyelectrolytes. Macromolecules 2000, 33, 4213–4219. 10.1021/ma991645q.

[ref17] Elzbieciak M.; Kolasińska M.; Zapotoczny S.; Krastev R.; Nowakowska M.; Warszyński P. Nonlinear Growth of Multilayer Films Formed from Weak Polyelectrolytes. Colloids Surf., A 2009, 343, 89–95. 10.1016/j.colsurfa.2009.01.034. 19437787

[ref18] Lundin M.; Solaqa F.; Thormann E.; Macakova L.; Blomberg E. Layer-by-Layer Assemblies of Chitosan and Heparin: Effect of Solution Ionic Strength and PH. Langmuir 2011, 27, 7537–7548. 10.1021/la200441u. 21574585

[ref19] Steitz R.; Jaeger W.; von Klitzing R. Influence of Charge Density and Ionic Strength on the Multilayer Formation of Strong Polyelectrolytes. Langmuir 2001, 17, 4471–4474. 10.1021/la010168d.

[ref20] McAloney R. A.; Sinyor M.; Dudnik V.; Cynthia Goh M. Atomic Force Microscopy Studies of Salt Effects on Polyelectrolyte Multilayer Film Morphology. Langmuir 2001, 17, 6655–6663. 10.1021/la010136q.

[ref21] Guzmán E.; Ritacco H.; Rubio J. E. F.; Rubio R. G.; Ortega F. Salt-Induced Changes in the Growth of Polyelectrolyte Layers of Poly(Diallyl-Dimethylammonium Chloride) and Poly(4-Styrene Sulfonate of Sodium). Soft Matter 2009, 5, 2130 10.1039/b901193e.

[ref22] Tan H. L.; McMurdo M. J.; Pan G.; Van Patten P. G. Temperature Dependence of Polyelectrolyte Multilayer Assembly. Langmuir 2003, 19, 9311–9314. 10.1021/la035094f.

[ref23] Salomäki M.; Vinokurov I. A.; Kankare J. Effect of Temperature on the Buildup of Polyelectrolyte Multilayers. Langmuir 2005, 21, 11232–11240. 10.1021/la051600k. 16285796

[ref24] Büscher K.; Graf K.; Ahrens H.; Helm C. A. Influence of Adsorption Conditions on the Structure of Polyelectrolyte Multilayers. Langmuir 2002, 18, 3585–3591. 10.1021/la011682m.

[ref25] Dubas S. T.; Schlenoff J. B. Factors Controlling the Growth of Polyelectrolyte Multilayers. Macromolecules 1999, 32, 8153–8160. 10.1021/ma981927a.

[ref26] Poptoshev E.; Schoeler B.; Caruso F. Influence of Solvent Quality on the Growth of Polyelectrolyte Multilayers. Langmuir 2004, 20, 829–834. 10.1021/la035485u. 15773111

[ref27] Jukić J.; Korade K.; Milisav A.-M.; Marion I. D.; Kovačević D. Ion-Specific and Solvent Effects on PDADMA–PSS Complexation and Multilayer Formation. Colloids Interfaces 2021, 5, 38 10.3390/colloids5030038.

[ref28] Haitami A. E. El.; Martel D.; Ball V.; Nguyen H. C.; Gonthier E.; Voegel J.; Schaaf P.; Senger B. Effect of the Supporting Electrolyte Anion on the Thickness of PSS/PAH Multilayer Films and on Their Permeability to an Electroactive Probe. Langmuir 2009, 25, 2282–2289. 10.1021/la803534y. 19123805

[ref29] Buron C. C.; Filiâtre C.; Membrey F.; Bainier C.; Buisson L.; Charraut D.; Foissy A. Surface Morphology and Thickness of a Multilayer Film Composed of Strong and Weak Polyelectrolytes: Effect of the Number of Adsorbed Layers, Concentration and Type of Salts. Thin Solid Films 2009, 517, 2611–2617. 10.1016/j.tsf.2008.10.036.

[ref30] Dodoo S.; Steitz R.; Laschewsky A.; von Klitzing R. Effect of Ionic Strength and Type of Ions on the Structure of Water Swollen Polyelectrolyte Multilayers. Phys. Chem. Chem. Phys. 2011, 13, 10318–10325. 10.1039/c0cp01357a. 21523268

[ref31] Dressick W. J.; Wahl K. J.; Bassim N. D.; Stroud R. M.; Petrovykh D. Y. Divalent-Anion Salt Effects in Polyelectrolyte Multilayer Depositions. Langmuir 2012, 28, 15831–15843. 10.1021/la3033176. 23106264

[ref32] Andreeva T. D.; Hartmann H.; Taneva S. G.; Krastev R. Regulation of the Growth, Morphology, Mechanical Properties and Biocompatibility of Natural Polysaccharide-Based Multilayers by Hofmeister Anions. J. Mater. Chem. B 2016, 4, 7092–7100. 10.1039/C6TB01638C. 32263646

[ref33] Salopek J.; Sadžak A.; Kuzman D.; Požar J.; Kovačević D. Polyelectrolyte Multilayers on Silica Surfaces: Effect of Ionic Strength and Sodium Salt Type. Croat. Chem. Acta 2017, 90, 281–287. 10.5562/cca3179.

[ref34] Liu G.; Hou Y.; Xiao X.; Zhang G. Specific Anion Effects on the Growth of a Polyelectrolyte Multilayer in Single and Mixed Electrolyte Solutions Investigated with Quartz Crystal Microbalance. J. Phys. Chem. B 2010, 114, 9987–9993. 10.1021/jp1018263. 20684620

[ref35] Feldötö Z.; Varga I.; Blomberg E. Influence of Salt and Rinsing Protocol on the Structure of PAH/PSS Polyelectrolyte Multilayers. Langmuir 2010, 26, 17048–17057. 10.1021/la102351f. 20886835

[ref36] Kovačević D.; van der Burgh S.; de Keizer A.; Cohen Stuart M. A. Specific Ionic Effects on Weak Polyelectrolyte Multilayer Formation. J. Phys. Chem. B 2003, 107, 7998–8002. 10.1021/jp0273777.

[ref37] Mermut O.; Barrett C. J. Effects of Charge Density and Counterions on the Assembly of Polyelectrolyte Multilayers. J. Phys. Chem. B 2003, 107, 2525–2530. 10.1021/jp027278t.

[ref38] Salomäki M.; Tervasmäki P.; Areva S.; Kankare J. The Hofmeister Anion Effect and the Growth of Polyelectrolyte Multilayers. Langmuir 2004, 20, 3679–3683. 10.1021/la036328y. 15875399

[ref39] Salomäki M.; Laiho T.; Kankare J. Counteranion-Controlled Properties of Polyelectrolyte Multilayers. Macromolecules 2004, 37, 9585–9590. 10.1021/ma048701u.

[ref40] Ball V.; Voegel J.-C.; Schaaf P. Effect of Thiocyanate Counterion Condensation on Poly(Allylamine Hydrochloride) Chains on the Buildup and Permeability of Polystyrenesulfonate/Polyallylamine Polyelectrolyte Multilayers. Langmuir 2005, 21, 4129–4137. 10.1021/la047610n. 15835984

[ref41] Kharlampieva E.; Pristinski D.; Sukhishvili S. A. Hydrogen-Bonded Multilayers of Poly(Carboxybetaine)S. Macromolecules 2007, 40, 6967–6972. 10.1021/ma071152i.

[ref42] Salomäki M.; Kankare J. Specific Anion Effect in Swelling of Polyelectrolyte Multilayers. Macromolecules 2008, 41, 4423–4428. 10.1021/ma800315j.

[ref43] Wong J. E.; Zastrow H.; Jaeger W.; von Klitzing R. Specific Ion versus Electrostatic Effects on the Construction of Polyelectrolyte Multilayers. Langmuir 2009, 25, 14061–14070. 10.1021/la901673u. 19705859

[ref44] Zan X.; Hoagland D. A.; Wang T.; Peng B.; Su Z. Polyelectrolyte Uptake by PEMs: Impacts of Molecular Weight and Counterion. Polymer 2012, 53, 5109–5115. 10.1016/j.polymer.2012.09.011.

[ref45] Reid D. K.; Summers A.; O’Neal J.; Kavarthapu A. V.; Lutkenhaus J. L. Swelling and Thermal Transitions of Polyelectrolyte Multilayers in the Presence of Divalent Ions. Macromolecules 2016, 49, 5921–5930. 10.1021/acs.macromol.6b01164.

[ref46] Wei J.; Hoagland D. A.; Zhang G.; Su Z. Effect of Divalent Counterions on Polyelectrolyte Multilayer Properties. Macromolecules 2016, 49, 1790–1797. 10.1021/acs.macromol.5b02151.

[ref47] O’Neal J. T.; Dai E. Y.; Zhang Y.; Clark K. B.; Wilcox K. G.; George I. M.; Ramasamy N. E.; Enriquez D.; Batys P.; Sammalkorpi M.; Lutkenhaus J. L. QCM-D Investigation of Swelling Behavior of Layer-by-Layer Thin Films upon Exposure to Monovalent Ions. Langmuir 2018, 34, 999–1009. 10.1021/acs.langmuir.7b02836. 29131641

[ref48] Hofmeister F. Zur Lehre von Der Wirkung Der Salze. Arch. Exp. Pathol. Pharmakol. 1888, 24, 247–260. 10.1007/BF01918191.

[ref49] Leontidis E. Hofmeister Anion Effects on Surfactant Self-Assembly and the Formation of Mesoporous Solids. Curr. Opin. Colloid Interface Sci. 2002, 7, 81–91. 10.1016/S1359-0294(02)00010-9.

[ref50] Požar J.; Bohinc K.; Vlachy V.; Kovačević D. Ion-Specific and Charge Effects in Counterion Binding to Poly(Styrenesulfonate) Anions. Phys. Chem. Chem. Phys. 2011, 13, 15610–15618. 10.1039/c1cp21291e. 21792404

[ref51] Katana B.; Takács D.; Csapó E.; Szabó T.; Jamnik A.; Szilagyi I. Ion Specific Effects on the Stability of Halloysite Nanotube Colloids—Inorganic Salts versus Ionic Liquids. J. Phys. Chem. B 2020, 124, 9757–9765. 10.1021/acs.jpcb.0c07885. 33076658 PMC7660744

[ref52] Katana B.; Takács D.; Bobbink F. D.; Dyson P. J.; Alsharif N. B.; Tomšič M.; Szilagyi I. Masking Specific Effects of Ionic Liquid Constituents at the Solid–Liquid Interface by Surface Functionalization. Phys. Chem. Chem. Phys. 2020, 22, 24764–24770. 10.1039/D0CP02805C. 33107516

[ref53] Takács D.; Katana B.; Szerlauth A.; Sebők D.; Tomšič M.; Szilágyi I. Influence of Adsorption of Ionic Liquid Constituents on the Stability of Layered Double Hydroxide Colloids. Soft Matter 2021, 17, 9116–9124. 10.1039/D1SM01074C. 34569591

[ref54] Para G.; Jarek E.; Warszynski P. The Hofmeister Series Effect in Adsorption of Cationic Surfactants—Theoretical Description and Experimental Results. Adv. Colloid Interface Sci. 2006, 122, 39–55. 10.1016/j.cis.2006.06.017. 16905112

[ref55] Aroti A.; Leontidis E.; Maltseva E.; Brezesinski G. Effects of Hofmeister Anions on DPPC Langmuir Monolayers at the Air–Water Interface. J. Phys. Chem. B 2004, 108, 15238–15245. 10.1021/jp0481512.

[ref56] Aroti A.; Leontidis E.; Dubois M.; Zemb T. Effects of Monovalent Anions of the Hofmeister Series on DPPC Lipid Bilayers Part I: Swelling and In-Plane Equations of State. Biophys. J. 2007, 93, 1580–1590. 10.1529/biophysj.106.094482. 17496051 PMC1948043

[ref57] Leontidis E.; Aroti A.; Belloni L. Liquid Expanded Monolayers of Lipids As Model Systems to Understand the Anionic Hofmeister Series: 1. A Tale of Models. J. Phys. Chem. B 2009, 113, 1447–1459. 10.1021/jp809443d. 19143560

[ref58] Apaydin K.; Laachachi A.; Bour J.; Toniazzo V.; Ruch D.; Ball V. Polyelectrolyte Multilayer Films Made from Polyallylamine and Short Polyphosphates: Influence of the Surface Treatment, Ionic Strength and Nature of the Electrolyte Solution. Colloids Surf., A 2012, 415, 274–280. 10.1016/j.colsurfa.2012.09.036.

[ref59] Koetz J.; Kosmella S. Polyelectrolytes and Nanoparticles, 1st ed.; Springer: Heidelberg, 2007.

[ref60] Choi J.; Rubner M. F. Influence of the Degree of Ionization on Weak Polyelectrolyte Multilayer Assembly. Macromolecules 2005, 38, 116–124. 10.1021/ma048596o.

[ref61] Neumann A. W.; Good R. J. Techniques of Measuring Contact Angles. In Surface and Colloid Science; Good R. J.; Stromberg R. R., Eds.; Springer: Boston, 1979; pp 31–91.

[ref62] de Río O. I.; Neumann A. W. Axisymmetric Drop Shape Analysis: Computational Methods for the Measurement of Interfacial Properties from the Shape and Dimensions of Pendant and Sessile Drops. J. Colloid Interface Sci. 1997, 196, 136–147. 10.1006/jcis.1997.5214. 9792739

[ref63] Jellison G. E. Jr. Optical Functions of Silicon Determined by Two-Channel Polarization Modulation Ellipsometry. Opt. Mater. 1992, 1, 41–47. 10.1016/0925-3467(92)90015-F.

[ref64] Ciddor P. E. Refractive Index of Air: New Equations for the Visible and near Infrared. Appl. Opt. 1996, 35, 1566 10.1364/AO.35.001566. 21085275

[ref65] Sun B.; Jewell C. M.; Fredin N. J.; Lynn D. M. Assembly of Multilayered Films Using Well-Defined, End-Labeled Poly(Acrylic Acid): Influence of Molecular Weight on Exponential Growth in a Synthetic Weak Polyelectrolyte System. Langmuir 2007, 23, 8452–8459. 10.1021/la7010875. 17616162

[ref66] Jukić J.; Kovačević D.; Cindro N.; Fink R.; Oder M.; Milisav A.-M.; Požar J. Predicting the Outcomes of Interpolyelectrolyte Neutralization at Surfaces on the Basis of Complexation Experiments and Vice Versa. Soft Matter 2022, 18, 744–754. 10.1039/D1SM01308D. 34927650

[ref67] Chen W.; McCarthy T. J. Layer-by-Layer Deposition: A Tool for Polymer Surface Modification. Macromolecules 1997, 30, 78–86. 10.1021/ma961096d.

[ref68] Yoo D.; Shiratori S. S.; Rubner M. F. Controlling Bilayer Composition and Surface Wettability of Sequentially Adsorbed Multilayers of Weak Polyelectrolytes. Macromolecules 1998, 31, 4309–4318. 10.1021/ma9800360.

[ref69] Wong J. E.; Rehfeldt F.; Hänni P.; Tanaka M.; Klitzing R. V. Swelling Behavior of Polyelectrolyte Multilayers in Saturated Water Vapor. Macromolecules 2004, 37, 7285–7289. 10.1021/ma0351930.

[ref70] Elzbieciak M.; Kolasinska M.; Warszynski P. Characteristics of Polyelectrolyte Multilayers: The Effect of Polyion Charge on Thickness and Wetting Properties. Colloids Surf., A 2008, 321, 258–261. 10.1016/j.colsurfa.2008.01.036.

[ref71] Ferreira A. M.; Gentile P.; Toumpaniari S.; Ciardelli G.; Birch M. A. Impact of Collagen/Heparin Multilayers for Regulating Bone Cellular Functions. ACS Appl. Mater. Interfaces 2016, 8, 29923–29932. 10.1021/acsami.6b09241. 27762547

[ref72] Wasilewska M.; Adamczyk Z.; Sadowska M.; Boulmedais F.; Cieśla M. Mechanisms of Fibrinogen Adsorption on Silica Sensors at Various PHs: Experiments and Theoretical Modeling. Langmuir 2019, 35, 11275–11284. 10.1021/acs.langmuir.9b01341. 31394033

[ref73] Fujita S.; Shiratori S. The Initial Growth of Ultra-Thin Films Fabricated by a Weak Polyelectrolyte Layer-by-Layer Adsorption Process. Nanotechnology 2005, 16, 1821–1827. 10.1088/0957-4484/16/9/068.

[ref74] Kremer T.; Kovačević D.; Salopek J.; Požar J. Conditions Leading to Polyelectrolyte Complex Overcharging in Solution: Complexation of Poly(Acrylate) Anion with Poly(Allylammonium) Cation. Macromolecules 2016, 49, 8672–8685. 10.1021/acs.macromol.6b01892.

[ref75] Batys P.; Kivistö S.; Lalwani S. M.; Lutkenhaus J. L.; Sammalkorpi M. Comparing Water-Mediated Hydrogen-Bonding in Different Polyelectrolyte Complexes. Soft Matter 2019, 15, 7823–7831. 10.1039/C9SM01193E. 31524209

[ref76] Wang L.; Lin Y.; Su Z. Counterion Exchange at the Surface of Polyelectrolyte Multilayer Film for Wettability Modulation. Soft Matter 2009, 5, 2072 10.1039/b900638a.

[ref77] Wenzel R. N. Resistance of Solid Surfaces to Wetting by Water. Ind. Eng. Chem. 1936, 28, 988–994. 10.1021/ie50320a024.

[ref78] Schlenoff J. B.; Ly H.; Li M. Charge and Mass Balance in Polyelectrolyte Multilayers. J. Am. Chem. Soc. 1998, 120, 7626–7634. 10.1021/ja980350.

[ref79] Schlenoff J. B.; Rmaile A. H.; Bucur C. B. Hydration Contributions to Association in Polyelectrolyte Multilayers and Complexes: Visualizing Hydrophobicity. J. Am. Chem. Soc. 2008, 130, 13589–13597. 10.1021/ja802054k. 18798621

[ref80] Farhat T.; Yassin G.; Dubas S. T.; Schlenoff J. B. Water and Ion Pairing in Polyelectrolyte Multilayers. Langmuir 1999, 15, 6621–6623. 10.1021/la990631a.

[ref81] Vidyasagar A.; Sung C.; Losensky K.; Lutkenhaus J. L. PH-Dependent Thermal Transitions in Hydrated Layer-by-Layer Assemblies Containing Weak Polyelectrolytes. Macromolecules 2012, 45, 9169–9176. 10.1021/ma3020454.

[ref82] Shao L.; Lutkenhaus J. L. Thermochemical Properties of Free-Standing Electrostatic Layer-by-Layer Assemblies Containing Poly(Allylamine Hydrochloride) and Poly(Acrylic Acid). Soft Matter 2010, 6, 3363–3369. 10.1039/c0sm00082e.

[ref83] Jang W. S.; Jensen A. T.; Lutkenhaus J. L. Confinement Effects on Cross-Linking within Electrostatic Layer-by-Layer Assemblies Containing Poly(Allylamine Hydrochloride) and Poly(Acrylic Acid). Macromolecules 2010, 43, 9473–9479. 10.1021/ma102043d.

[ref84] Dodoo S.; Balzer B. N.; Hugel T.; Laschewsky A.; Von Klitzing R. Effect of Ionic Strength and Layer Number on Swelling of Polyelectrolyte Multilayers in Water Vapour. Soft Mater. 2013, 11, 157–164. 10.1080/1539445X.2011.607203.

[ref85] Zerball M.; Laschewsky A.; von Klitzing R. Swelling of Polyelectrolyte Multilayers: The Relation Between, Surface and Bulk Characteristics. J. Phys. Chem. B 2015, 119, 11879–11886. 10.1021/acs.jpcb.5b04350. 26267270

[ref86] Jomaa H. W.; Schlenoff J. B. Salt-Induced Polyelectrolyte Interdiffusion in Multilayered Films: A Neutron Reflectivity Study. Macromolecules 2005, 38, 8473–8480. 10.1021/ma050072g.

[ref87] Fares H. M.; Schlenoff J. B. Diffusion of Sites versus Polymers in Polyelectrolyte Complexes and Multilayers. J. Am. Chem. Soc. 2017, 139, 14656–14667. 10.1021/jacs.7b07905. 28981268

[ref88] Abbett R. L.; Chen Y.; Schlenoff J. B. Self-Exchange of Polyelectrolyte in Multilayers: Diffusion as a Function of Salt Concentration and Temperature. Macromolecules 2021, 54, 9522–9531. 10.1021/acs.macromol.1c01464.

[ref89] Ghostine R. A.; Jisr R. M.; Lehaf A.; Schlenoff J. B. Roughness and Salt Annealing in a Polyelectrolyte Multilayer. Langmuir 2013, 29, 11742–11750. 10.1021/la401632x. 24004344

[ref90] Bucur C. B.; Sui Z.; Schlenoff J. B. Ideal Mixing in Polyelectrolyte Complexes and Multilayers: Entropy Driven Assembly. J. Am. Chem. Soc. 2006, 128, 13690–13691. 10.1021/ja064532c. 17044688

[ref91] Gustafsson E.; Hedberg J.; Larsson P. A.; Wågberg L.; Johnson C. M. Vibrational Sum Frequency Spectroscopy on Polyelectrolyte Multilayers: Effect of Molecular Surface Structure on Macroscopic Wetting Properties. Langmuir 2015, 31, 4435–4442. 10.1021/la5046207. 25859709

[ref92] Hariri H. H.; Lehaf A. M.; Schlenoff J. B. Mechanical Properties of Osmotically Stressed Polyelectrolyte Complexes and Multilayers: Water as a Plasticizer. Macromolecules 2012, 45, 9364–9372. 10.1021/ma302055m.

[ref93] Zhang R.; Zhang Y.; Antila H. S.; Lutkenhaus J. L.; Sammalkorpi M. Role of Salt and Water in the Plasticization of PDAC/PSS Polyelectrolyte Assemblies. J. Phys. Chem. B 2017, 121, 322–333. 10.1021/acs.jpcb.6b12315. 27960054

