# Supplementary material for: Suppressing the Hofmeister Anion Effect by Thermal Annealing of Thin-Film Multilayers Made of Weak Polyelectrolytes
Source: Macromolecules. 2022 Oct 26;55(21):9571–82. doi: 10.1021/acs.macromol.2c01517 (PMC9661731; doi:10.1021/acs.macromol.2c01517)
Supplement: Supplementary file 1 — ma2c01517_si_001.pdf [file ma2c01517_si_001.pdf]

Supporting Information for

Suppressing the Hofmeister anion effect by thermal annealing of thin film multilayers made of weak polyelectrolytes

Tin Klačić,[a,*] Klemen Bohinc,[b] and Davor Kovačević[a,*]

[a] Division of Physical Chemistry, Department of Chemistry, Faculty of Science, University of Zagreb, Horvatovac 102a, 10000 Zagreb, Croatia

[b] Faculty of Health Sciences, University of Ljubljana, Zdravstvena pot 5, 1000 Ljubljana, Slovenia

[*] Corresponding authors:

E-mail address: davor.kovacevic@chem.pmf.hr (D. Kovačević), tklacic@chem.pmf.hr (T. Klačić)

This PDF file includes: 1) AFM film thickness measurements

2) Figures

3) Tables

1) AFM film thickness measurements

Samples were prepared for AFM measurement of total PEM thickness by gently scribing $(PAH/PAA)_5$ films deposited onto silicon substrate with sharp stainless steel microscope tweezers (model: EM-Tec 3C.AM, Micro to Nano). In this way, a step edge boundary was created. Then the measurements were made by scanning the AFM tip across step edge boundary in a soft tapping mode. At first a 25×25 μm^2 overview of the PEM was imaged and then a 5×5 μm^2 image in edge area of the previously scanned 25×25 μm^2 image was taken. A representative example of such AFM image accompanied by a height profile is shown in Figure 5 (main text). Finally, the total film thickness was calculated using sophisticated terraces feature in Gwyddion 2.54 software after ordinary first-order flattening routine in NanoScope Analyses 2.0 program. As Gwyddion 2.54 software shows a result in the form of an average film thickness and its standard deviation, they are reported in the manuscript.

2) Figures

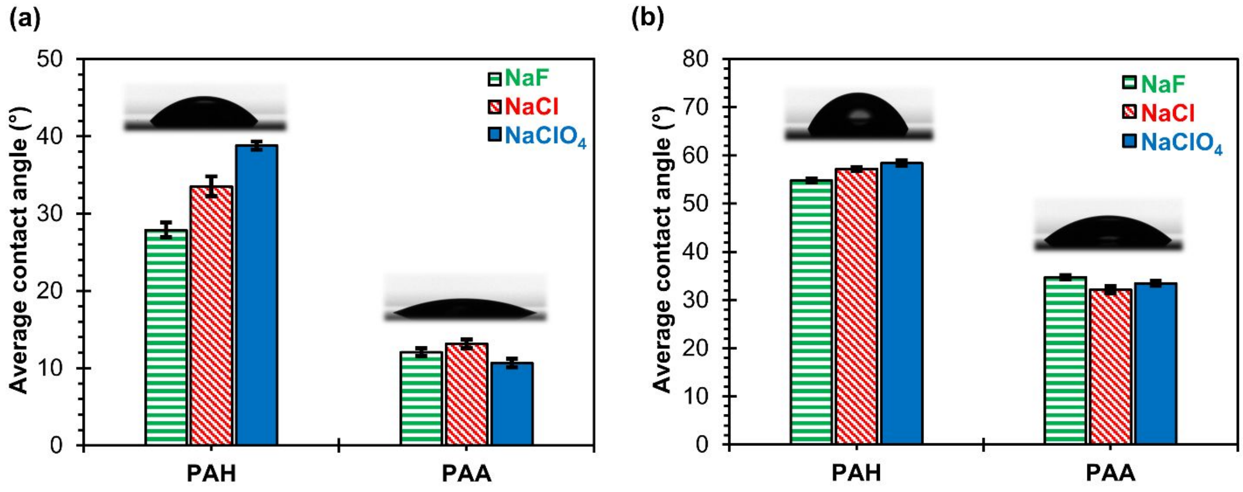

Figure S1. Average water contact angles of PAH and PAA layers prepared by the LbL method from dipping solutions with different sodium salts and drying of the films with: (a) nitrogen and (b) nitrogen and for 30 minutes at 60 °C in the dryer. The average contact angles of PAH and PAA were calculated as the arithmetic means of the contact angles of the last three polycation and polyanion layers, respectively. Photo excerpts show water droplets on the surface of the 9th and 10th layers prepared from $NaClO_4$.

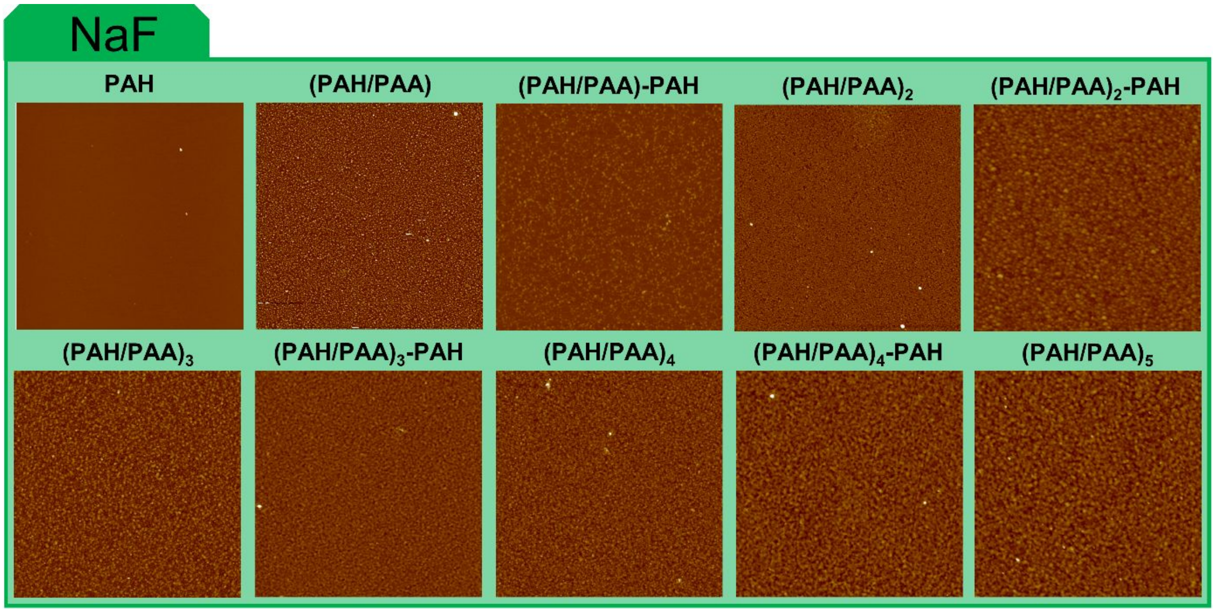

Figure S2. AFM images of unheated PAH/PAA films with different number of layers. Multilayers were assembled from 0.01 M polyelectrolyte solutions with 0.1 M NaF as a background electrolyte. Images have a scan size of 5 × 5 μm^2 and the *z*-scale is set to 30 nm.

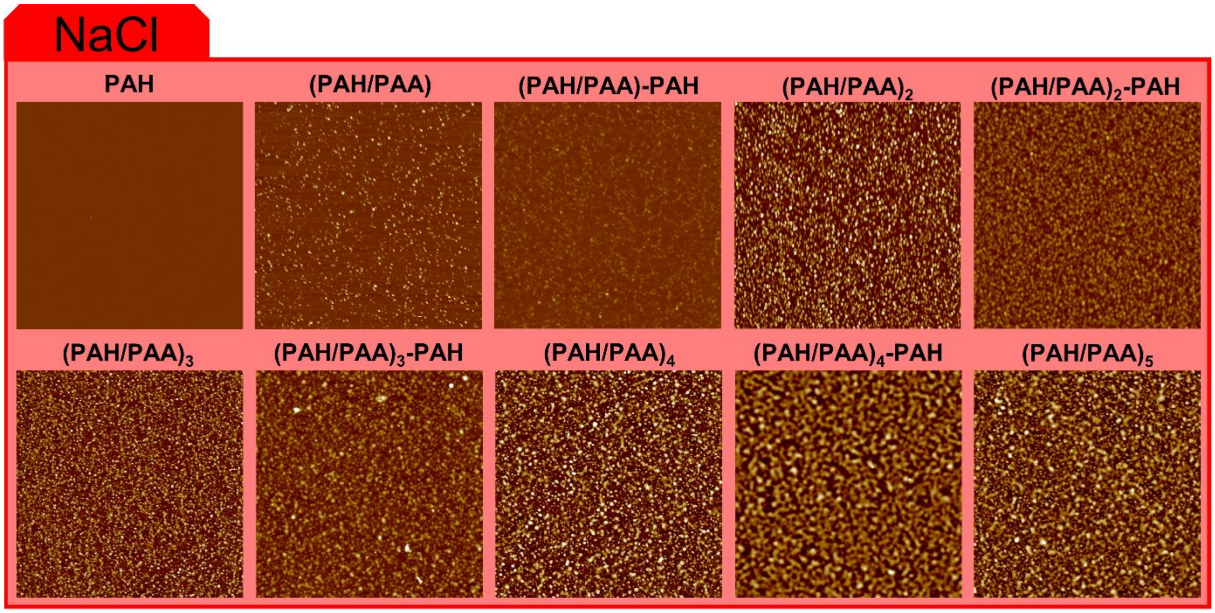

Figure S3. AFM images of unheated PAH/PAA films with different number of layers. Multilayers were assembled from 0.01 M polyelectrolyte solutions with 0.1 M NaCl as a background electrolyte. Images have a scan size of 5 × 5 μm^2 and the *z*-scale is set to 30 nm.

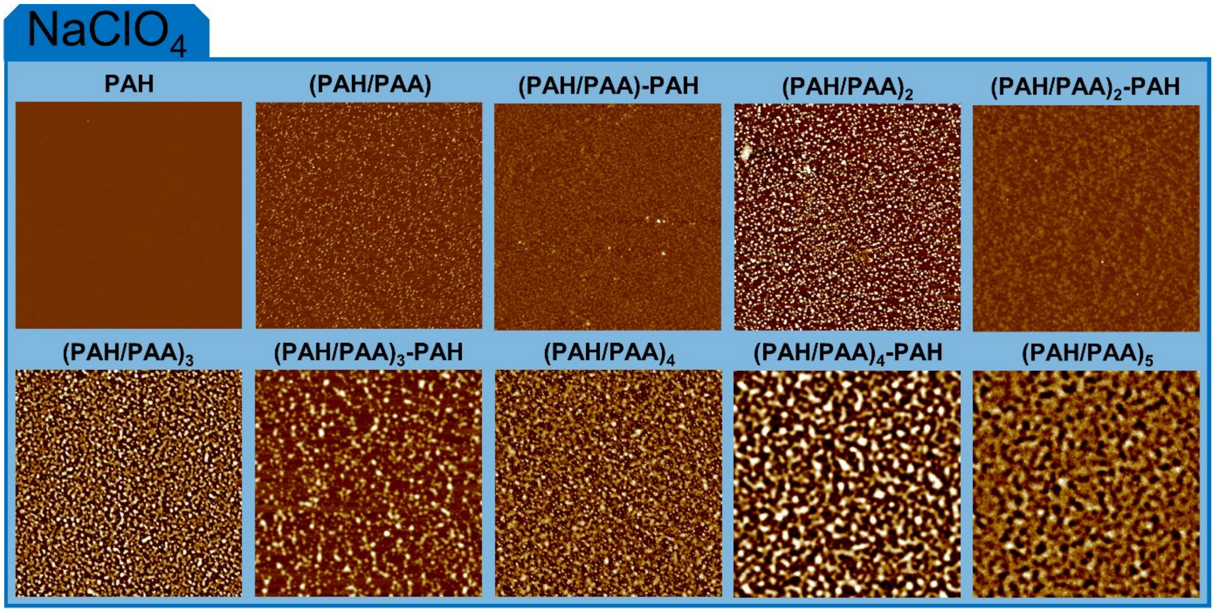

Figure S4. AFM images of unheated PAH/PAA films with different number of layers. Multilayers were assembled from 0.01 M polyelectrolyte solutions with 0.1 M $NaClO_4$ as a background electrolyte. Images have a scan size of 5 × 5 μm^2 and the *z*-scale is set to 30 nm.

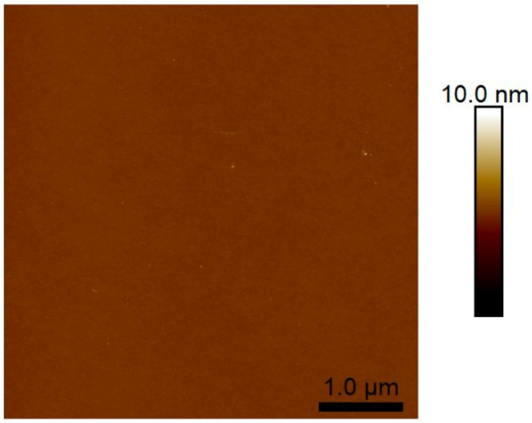

Figure S5. AFM image of silicon wafer surface. Image was acquired in ambient conditions using AFM contact mode and ScanAsyst-air probe (Bruker).

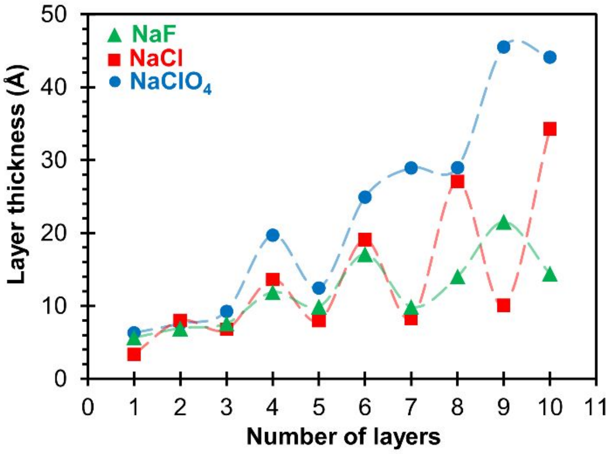

Figure S6. Thickness of each PAH and PAA layer during the multilayer build-up obtained by ellipsometer as a difference between thickness of film with *N* and *N*−1 layers. Multilayers were assembled on Si wafer at pH = 7.0 from 0.01 M polyelectrolyte solutions in different background sodium salts (c = 0.1 M). Heating was not applied during the layer-by-layer process. Odd numbers represent films with PAH as the outermost layer, whereas even number films have PAA as the outermost layer. Dashed lines have no physical meaning and were added as a guide to the eye.

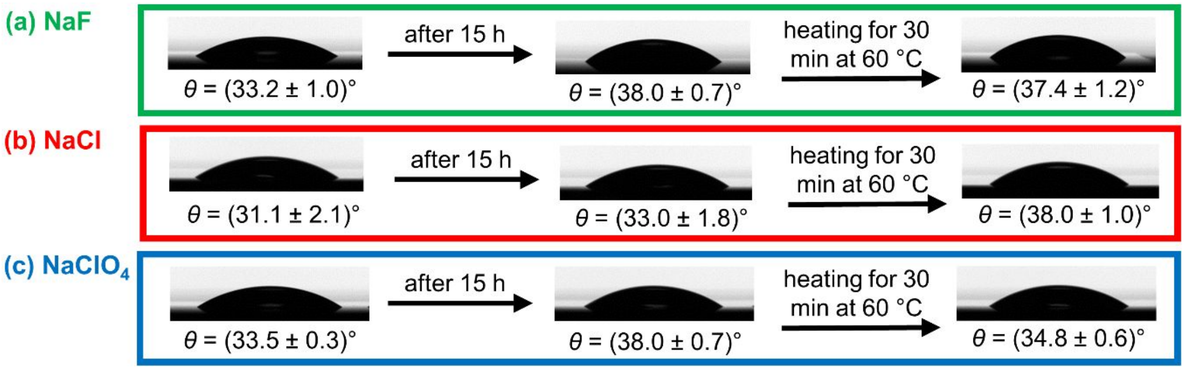

Figure S7. Advancing water contact angle (and standard error) of heated $(PAH/PAA)_5$ multilayers measured at (24 ± 2) °C and relative humidity 30 − 50% immediately after preparation, after 15 hours and after additional heating for 30 minutes at 60 °C. PEMs were prepared from polyelectrolyte solutions (c_m = 0.01 M) in 0.10 M supporting electrolyte containing NaF (a), NaCl (b), and $NaClO_4$ (c) at pH = 7.0. Photo excerpts show water droplets on multilayer surface.

3) Tables

Table S1. Total thickness of $(PAH/PAA)_5$ multilayer determined by ellipsometer and AFM. Films were deposited on Si wafer at pH = 7.0 from 0.01 M polyelectrolyte solutions in different background sodium salts (c = 0.1 M). Some of the samples were heated at 60 °C for 30 min after the drying step of LbL process.

	**AFM thickness (nm)**[a]		**Ellipsometric thickness (nm)**[b]	
**Salt**	**Unheated**	**Heated**	**Unheated**	**Heated**
NaF	10.8 ± 1.4	12.0 ± 1.2	11.9 ± 0.4	11.7 ± 1.2
NaCl	11.9 ± 3.0	13.7 ± 2.1	13.9 ± 1.4	15.4 ± 1.5
$NaClO_4$	19.8 ± 5.5	21.6 ± 1.1	22.8 ± 0.6	25.3 ± 3.0

[a] Average film thickness and standard deviation calculated from AFM measurement as described in Section 1 of Supporting Information.

[b] Average thickness and standard deviation of ellipsometric measurements at 10 different locations on each sample
